# Phytoplankton Temporal Strategies Increase Entropy Production in a Marine Food Web Model

**DOI:** 10.3390/e22111249

**Published:** 2020-11-03

**Authors:** Joseph J. Vallino, Ioannis Tsakalakis

**Affiliations:** 1 Marine Biological Laboratory, Woods Hole, MA 02543, USA; itsakalakis@mbl.edu; 2 Department of Earth, Atmosphere and Planetary Sciences, Massachusetts Institute of Technology, Cambridge, MA 02139, USA

**Keywords:** maximum entropy production, trait-based modeling, temporal strategy, circadian rhythm, biogeochemistry, food web model

## Abstract

We develop a trait-based model founded on the hypothesis that biological systems evolve and organize to maximize entropy production by dissipating chemical and electromagnetic free energy over longer time scales than abiotic processes by implementing temporal strategies. A marine food web consisting of phytoplankton, bacteria, and consumer functional groups is used to explore how temporal strategies, or the lack thereof, change entropy production in a shallow pond that receives a continuous flow of reduced organic carbon plus inorganic nitrogen and illumination from solar radiation with diel and seasonal dynamics. Results show that a temporal strategy that employs an explicit circadian clock produces more entropy than a passive strategy that uses internal carbon storage or a balanced growth strategy that requires phytoplankton to grow with fixed stoichiometry. When the community is forced to operate at high specific growth rates near 2 d^−1^, the optimization-guided model selects for phytoplankton ecotypes that exhibit complementary for winter versus summer environmental conditions to increase entropy production. We also present a new type of trait-based modeling where trait values are determined by maximizing entropy production rather than by random selection.

## 1. Introduction

The maximum entropy production (MEP) principle postulates that steady state, non-equilibrium systems with many degrees of freedom will likely organize to maximize entropy production, or equivalently, maximize the dissipation rate of Gibbs free energy (hence forth free energy) [1,2,3]. MEP applications can be traced back to at least Paltridge [4], and perhaps to even Lotka [5], and MEP theory appears to have multiple origins [4,6,7,8], but over the last decade and a half there has been renewed interest in extending MEP theory as well as its applications [9,10]. Since MEP makes no distinction between abiotic or biotic systems, MEP research has been wide ranging, from crystal growth [11], Rayleigh–Benard convection [12], and phase transitions [13] to Earth’s hydrologic cycle [14], ocean circulation [15], ecology [16], biogeochemistry [17], and evolution [18] to name just a few. The MEP approach has garnered interest in systems where classical reductionist modeling is difficult to implement due to insufficient information or understanding, such as turbulent flow and living systems that are governed by self-organization. For systems where we have good reductionist understanding and modeling capabilities (i.e., Newtonian physics), MEP may provide some benefit from a wholistic perspective, but otherwise may not be of much benefit.

One of the important uncertainties regarding MEP theory concerns its applicability to non-steady state systems for which no theory currently exists. There have been some attempts to extend MEP theory to non-steady state systems [19,20], but no consensus has emerged. Consequently, we have chosen a different approach to exploring MEP in non-steady state systems based on numerical modeling of the chemistry catalyzed by microbial systems and comparing modeling results to observations or our basic understanding of these systems. If MEP-based models show good predictive capabilities, then the likelihood that MEP governs their function and evolution is supported. This is particularly relevant for biological systems as we have yet to develop good predictive understanding of their behavior due to their complexity and self-organizing capabilities. Bacteria and archaea have evolved the capability to exploit redox chemical potentials found in numerous environments on Earth, such as oxidation of hydrogen sulfide or ammonium with oxygen while fixing carbon dioxide (chemolithoautotrophy), oxidation of reduced organic carbon with sulfate or nitrate (anerobic metabolism), and oxidation of hydrogen with carbon dioxide to synthesize methane (methanogenesis) to name just a few [21]. All these exergonic reactions occur abiotically, but the presence of biology can increase reaction rates and associated dissipation of free energy by many orders of magnitude. Modeling this biogeochemistry using reductionism is challenging because a litter of water contains 10^9^ or more individuals, thousands to perhaps tens of thousands of different species [22], all of which are subject to predation and viral attack; soils and sediments are approximately 1000 times more complex. While it may not be possible to model all the details of these communities and the associated chemistry they catalyze, MEP provides an opportunity for prediction, assuming living systems evolve, organize, and function to dissipate free energy. If MEP theory does not explain microbial systems, there seems little expectation that it would be useful in describing biogeochemistry of higher trophic levels where the theory of large numbers is even less applicable [23], so microbial systems are a good place to test MEP-based hypotheses.

Our approach to modeling microbial biogeochemistry uses an abstracted distributed metabolic network [24] to represent the possible reactions a microbial community as a whole can catalyze, with emphasis placed on compounds found in the environment rather than those found within a cell. The reactions included in the network are divided into those that release Gibbs free energy (exergonic or catabolic reactions) and those associated with biomass synthesis (endergonic or anabolic reactions), where biomass is considered more as a catalyst than an organism. In fact, the catalyst associated with a given redox reaction can represent numerous species capable of conducting the reaction but are not distinguished in the model. Initial research on this approach focusing on non-steady state systems revealed that the time scale over which entropy is maximized changes the solution significantly [25]. In particular, if entropy production (EP) is maximized instantaneously then no biomass is synthesized, but the solution does exhibit characteristics of abiotic processes, while if EP is maximized over a finite time interval, then the solutions are consistent with the actions of biology, and more entropy can be produced. Based on this initial work [17,26,27], our current working hypothesis is that living systems use information acquired by evolution, stored in the genome, to maximize destruction of free energy over time scales where prediction fidelity is sufficient, which may depend on environmental dynamics and the ability of biology to measure its surrounding [28]. Anticipatory control can lead to greater EP over time, which differs from abiotic systems that maximize EP instantaneously, such as fire or a rock rolling down a hill. That is, abiotic systems follow steepest descent trajectories down free energy surfaces, while biological systems follow different pathways that lead to greater EP over time. This working hypothesis can be extended to space as well [29], where abiotic systems maximize local EP and biological systems maximize EP integrated over space by coordinating function via communication, such as quorum sensing, but we will not consider space in this manuscript.

One of the hallmarks of biology is the evolution of temporal strategies, such as circadian rhythms [30,31], food and resource storage [32,33], anticipatory control [34,35] and dormancy [36,37], which are consistent with our MEP hypothesis regarding abiotic versus biotic systems; however, such temporal strategies are seldom incorporated into models describing biogeochemistry of microbial processes. The objectives of this manuscript are to (1) illustrate an approach for incorporating temporal strategies into biogeochemical models, (2) test a new approach that replaces our previously developed optimal control approach with trait-based modeling where trait values are determined by MEP optimization, and (3) show that inclusion of temporal strategies leads to greater EP. We use a marine microbial food web as an example system that consists of photoautotrophs (aka, phytoplankton), aerobic heterotrophic bacteria that consume dissolved organic matter, and general consumers that can prey on both phototrophs and heterotrophs, as well as cannibalize themselves.

## 2. Model Description

The overall objective of the model is to explore how the addition of phytoplankton temporal strategies alter entropy production (EP) in marine planktonic food webs that dissipate electromagnetic and chemical free energy from solar radiation and reduced organic carbon inputs, such as glucose. We also investigate a new hybrid approach that uses EP maximization to set trait values in a trait-based modeling approach. The details and governing equations for the model are described in detail in Supplementary Materials, but key aspects of the model are described here. Double bracket notation will be used to denote a variable’s units in the model, such as ⟦m⟧ for meters.

### 2.1. Entropy Production

As discussed elsewhere [5,38,39], EP in this manuscript refers solely to irreversible processes and does not refer to system or state entropy, which traditionally is represented by the symbol *S* $⟦JK^{-1}⟧$. We follow convention here and use $\dot{\sigma }⟦J\;d^{-1}\;K^{-1}⟧$ to represent EP from irreversible processes, and $\sigma ⟦J\;K^{-1}⟧$ to represent cumulative entropy produced from irreversible processes over a specified time interval, Δt, so that $\sigma =\int_{t}^{t+\Delta t}\dot{\sigma }d\tau$. Thermodynamic entropy production is the destruction of Gibbs free energy; contrary to popular believe, system order contributes little to $\dot{\sigma }$ for biological systems or the chemistry they catalyze [40]. For chemical and biological systems, entropy production is readily calculated from the product of the reaction rate, $r⟦mmol\;m^{-3}\;d^{-1}⟧$, times the Gibbs free energy of reaction, $\Delta_{r}G⟦J\;mmol^{-1}⟧$, divided by temperature, as given by,

(1) $$\dot{\sigma }=-\frac{1}{T}r\Delta_{r}G$$

For non-steady state systems, maximizing σ depends on the time interval, Δt, chosen. For instance, in biological systems if entropy production is maximized instantaneously, then no organismal growth can occur. Consider a system that starts with some bacterial biomass present, along with some chemical potential, such as glucose plus oxygen. The maximum instantaneous EP is attained by maximizing the rate of glucose oxidation, as any co-synthesis of biomass would reduce EP. Furthermore, if a bacterial consumer is present, then instantaneous EP can be increased further by oxidizing the bacteria as well. Hence, under instantaneous EP, all chemical free energy gets destroyed, which is analogous to fire. However, if EP is maximized over some time interval, such a day, then synthesizing biomass can result in greater EP over the interval because reaction rate is proportional to biomass concentration, and entropy production is proportional to reaction rate, as given by Equation (1). Mathematically, this inequality can be expressed as,

(2) $$\max\left(\overset{t+\Delta t}{\underset{t}{\int }}\dot{\sigma }\left(\tau \right)d\tau \right)\geq \overset{t+\Delta t}{\underset{t}{\int }}\max\;\left(\dot{\sigma }\left(\tau \right)\right)d\tau ,$$

where the left-hand side of Equation (2) represents biological processes and the temporal strategies organisms implement, while the right-hand side of Equation (2) represents instantaneous, or steepest decent trajectory, abiotic systems follow, but see [5,25,26,27] for further discussion. Equation (2) is the fundamental hypothesis of the model.

### 2.2. Metabolic Reactions

**Figure 1:**
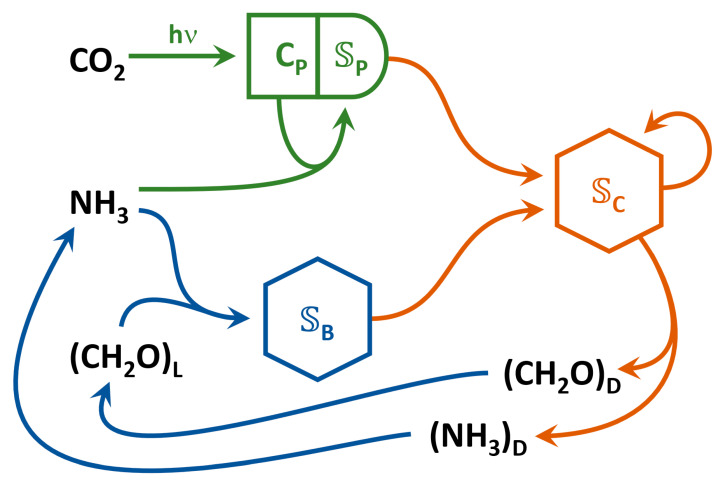
Food web structure used in the marine plankton model consisting of three function groups representing phytoplankton, ${𝕊}_{P}$, with their internal CH_2_O storage, $C_{P}$, bacteria, ${𝕊}_{B}$, and consumers, ${𝕊}_{C}$. Colored lines correspond to reactions a functional group is capable of catalyzing. The thermodynamic properties of glucose (unit carbon basis) is used to represent labile and detrital carbon, ${\left(CH_{2}O\right)}_{L}$ and ${\left(CH_{2}O\right)}_{D}$, respectively, while ammonium is used to represent detrital nitrogen, ${\left(NH_{3}\right)}_{D}$.

The model, derived in part from previous work focused on the biogeochemistry in a meromictic pond [17], consists of three functional groups: phytoplankton, ${𝕊}_{P}⟦mmol\;m^{-3}⟧$, which intercept high-frequency photons, $\gamma_{H}⟦mmol-\gamma ⟧$, and fix CO_2_ producing O_2_; bacteria, ${𝕊}_{B}$, that consume labile organic carbon, $C_{L}$, and decompose detrital organic carbon, $C_{D}$, and nitrogen, $N_{D}$, into labile constituents; consumers, ${𝕊}_{C}$, that prey on phytoplankton and bacteria, as well as themselves and produce detrital organic carbon and nitrogen (Figure 1). Unlike our previous models that contained just one state variable for each functional group, in the trait-based approach, there are $n_{P}$, $n_{B}$, and $n_{C}$ instances or ecotypes of ${𝕊}_{P\left\{i\right\}}$, ${𝕊}_{B\left\{i\right\}}$, and ${𝕊}_{C\left\{i\right\}}$, respectively, where a particular ecotype is distinguished using braces nomenclature, such as P{i}. The symbol 𝕊 represents biological Structure to emphasize its action as a reaction catalyst as opposed to the organismal centric view typically pursued in biology, and for simplicity the elemental composition for all 𝕊 is assumed to be the same, as given by $CH_{\alpha_{𝕊}}O_{\beta_{𝕊}}N_{\gamma_{𝕊}}P_{\delta_{𝕊}}$. The generalized reactions each functional group catalyzes are listed in Table 1, and the stoichiometrically balanced reactions are provided in Section S2.2 of the Supplementary Material. Two different kinetics expressions govern growth of phototrophs, ${𝕊}_{P}$, one of which is also used for heterotrophs, ${𝕊}_{B}$, ${𝕊}_{C}$, as described in Section 2.3 below.

To remove parameters whose values are largely unknown and poorly bounded, we formulate growth kinetics to depend on just two types of control variables whose values are determined over time to maximize EP, so that there are no parameters that require tuning. Consequently, the MEP approach requires very little information other than the biogeochemical reactions the community is capable of catalyzing and the constraints the environment places on the system, which is largely in the form of transport processes that govern free energy and resources input and output to the system. A metabolic reaction for a microorganism is constructed from a combination of two sub-reaction types, one associated with extraction of Gibbs free energy from either chemical reactions or electromagnetic radiation, and the other associated with biosynthesis of biological structure, 𝕊 (i.e., catalyst), driven by the extracted free energy. This representation derives from biochemistry where catabolic reactions produce ATP (adenosine triphosphate) to drive anabolic reactions forward. The two sub-reactions are combined to give a single reaction in the metabolic network, where the weighting between the catabolic and anabolic components of a reaction is controlled by a thermodynamic efficiency parameter, $\varepsilon_{\chi \left\{i\right\}}$.

Two reactions are used to describe the growth and metabolism of phototrophs. In the first reaction, $R_{1,P\left\{i\right\}}$, dissolved inorganic carbon and water, $H_{2}{\mathrm{CO}}_{3}$, are converted to internal stores of sugar, $C_{P\left\{i\right\}}$, with oxygen, $O_{2}$, produced as a byproduct. Because this reaction is thermodynamically uphill $\left(\Delta_{r}G>0\right)$, it is driven forward by free energy obtained by the capture of high frequency photons, $\gamma_{H}$, in photosynthetic active radiation (PAR) as given by,

(3) $$\varepsilon_{P\left\{i\right\}}H_{2}{\mathrm{CO}}_{3}+n_{1,P\left\{i\right\}}\gamma_{H}\overset{\Omega_{1.P}\left\{i\right\}{𝕊}_{P\left\{i\right\}}}{\to }\varepsilon_{P\left\{i\right\}}\left(C_{P\left\{i\right\}}+O_{2}\right),$$

where the thermodynamic efficiency variable, $\varepsilon_{P\left\{i\right\}}$, determines the fraction of free energy derived from photons (catabolic reaction) that is used to drive the fixation of carbon dioxide (CO_2_) into sugar,$\;C_{P\left\{i\right\}}$, and $O_{2}$ (anabolic reaction). The term above the arrow, $\Omega_{1.P\left\{i\right\}}{𝕊}_{P\left\{i\right\}}\;$, indicates the reaction is catalyzed by phytoplankton, ${𝕊}_{P\left\{i\right\}}$, but only by the faction of phytoplankton biomass that is allocated to photosynthesis specified by the variable $\Omega_{1.P\left\{i\right\}}$. The stoichiometric coefficient $n_{1,P\left\{i\right\}}$ is the moles of photons needed to reversibly fix one mole of carbon dioxide and is calculated from the free energy of photons and the free energy of reaction for CO_2_ fixation accounting for the reaction quotient under the prevailing environmental conditions (Equations (S4)–(S6)). Consequently, when $\varepsilon_{P\left\{i\right\}}$ equals 1, free energy is conserved, no entropy is produced, and the Gibbs free energy of reaction for $R_{1,P\left\{i\right\}}$, defined as $\Delta_{r}G_{1,P\left\{i\right\}}$, equals 0. That is, the reaction is at equilibrium so the net reaction rate, defined by $r_{1,P\left\{i\right\}}$, is also 0 (however, see Supplementary Materials S2.2.1 on photon free energy dissipation by reaction versus particles). At the other extreme, when $\varepsilon_{P\left\{i\right\}}$ equals 0, all the photon free energy is dissipated as heat, no CO_2_ is fixed, and entropy production based on Equation (1) is maximized, where the rate of reaction, $r_{1,P\left\{i\right\}}$, is based on the rate of photon interception described in Section 2.3 below.

The second reaction catalyzed by phytoplankton, $R_{2,P\left\{i\right\}}$, converts the fixed carbon, $C_{P\left\{i\right\}}$, plus resource needed for phytoplankton biosynthesis, such ammonium ${\mathrm{NH}}_{3}$, into biomass (anabolic reaction) as given by,

(4) $$C_{P\left\{i\right\}}+\varepsilon_{P\left\{i\right\}}\gamma_{𝕊}{\mathrm{NH}}_{3}+a_{2,P}^{A}\left(1-\varepsilon_{P\left\{i\right\}}\right)O_{2}\overset{\Omega_{2.P\left\{i\right\}}{𝕊}_{P\left\{i\right\}}}{\to }\varepsilon_{P\left\{i\right\}}{𝕊}_{P\left\{i\right\}}+b_{2,P}^{A}H_{2}O+\left(1-\varepsilon_{P}\right){\mathrm{CO}}_{2},$$

where $\gamma_{𝕊}$ is defined by the composition of $𝕊\;(CH_{\alpha_{𝕊}}O_{\beta_{𝕊}}N_{\gamma_{𝕊}}P_{\delta_{𝕊}}$) and $a_{2,P}^{A}$ and $b_{2,P}^{A}$ are stoichiometric coefficients needed to balance O and H that also depend on the composition of 𝕊 (Equations (S17) and (S18)). Oxidation of $C_{P\left\{i\right\}}$ to CO_2_ and water is the catabolic reaction that drives biosynthesis, which is included in Equation (4), where again $\varepsilon_{P\left\{i\right\}}$ provides coupling between the two sub-reactions. When $\varepsilon_{P\left\{i\right\}}$ equals 1, $R_{2,P\left\{i\right\}}$ is formulated to be at equilibrium (see Equation (S16) for details), while when $\varepsilon_{P\left\{i\right\}}$ equals 0, $C_{P\left\{i\right\}}$ is completely combusted and all free energy is dissipated as heat resulting in maximum entropy production. The variable $\Omega_{2.P\left\{i\right\}}$ is the fraction of phytoplankton biomass allocated to catalyzing the biosynthesis reaction, $R_{2,P\left\{i\right\}}$, but since $\Omega_{1.P\left\{i\right\}}{𝕊}_{P\left\{i\right\}}+\Omega_{2.P\left\{i\right\}}{𝕊}_{P\left\{i\right\}}$ must sum to ${𝕊}_{P\left\{i\right\}}$, increasing the rate of biosynthesis by allocating more catalyst to $R_{2,P\left\{i\right\}}$ results in a decreased allocation of catalyst to CO_2_ fixation, $R_{1,P\left\{i\right\}}$, and vice versa for increasing catalyst allocation to $R_{1,P\left\{i\right\}}$. Note, the complete stoichiometry for reaction $R_{2,P\left\{i\right\}}$, Equation (S16), is also balanced so that when $\varepsilon_{P\left\{i\right\}}$ equals 1, $\Delta_{r}G_{2,P\left\{i\right\}}$ equals 0.

For chemotrophic bacteria and heterotrophic consumers, reactions are similar to that given by Equation (4), except more reactions are used to represent the functional groups (Table 1). For example, in addition to the equation for bacterial growth, $R_{1,B\left\{i\right\}}$, bacteria also have the capability to decompose recalcitrant detrital carbon, $C_{D}$, and nitrogen, $N_{D}$, into labile pools via $R_{2,B\left\{i\right\}}$ and $R_{3,B\left\{i\right\}}$, respectively (Table 1). The amount of catalyst allocated to the three reactions is governed by the control variables $\Omega_{1,B\left\{i\right\}}$, $\Omega_{2,B\left\{i\right\}}$ and $\Omega_{3,B\left\{i\right\}}$, respectively, but these control variables must sum to 1, so only two degrees of freedom are needed to determine the partitioning of ${𝕊}_{B\left\{i\right\}}$ to the three reactions. Gibbs free energy of reaction for the two decomposition reactions is calculated from the logarithm of the concentration differences between reactants and products, as the Gibbs free energy of formation is assumed to be equal for labile and recalcitrant C and N pools (see Equations (S42)–(S47)) in Supplementary Materials S2.2.2).

For the heterotrophic consumers, which can consume all prey including other consumers and themselves, each consumer has $n_{P}+n_{B}+n_{C}$ prey consuming reactions, where $\Omega_{\chi \left\{j\right\},C\left\{i\right\}}$ determines allocation of catalyst, ${𝕊}_{C\left\{i\right\}}$, to each prey, and χ{j} is either P{j}, B{j} or C{j}. Unlike phytoplankton and bacteria, it is assumed consumers allocate catalytic resources to prey based on the preys concentration relative to all prey they are allowed to consume, so reaction rates depend on a weighted version of $\Omega_{\chi \left\{j\right\},C\left\{i\right\}}$ denoted as $\omega_{\chi \left\{j\right\},C\left\{i\right\}}$ as defined by Equation (S57). The predation by consumers also produces labile nitrogen as well as recalcitrant carbon on nitrogen as a function of $\varepsilon_{C\left\{i\right\}}$, Equation (S51), which captures “sloppy feeding” and incomplete digestion [41]. Furthermore, when consumers prey on phytoplankton, it is assumed that the labile carbon they contain, $C_{P\left\{i\right\}}$, is combusted and not incorporated into catalyst but does contribute to the Gibbs free energy of reaction, as given by Equations (S32)–(S36). Reaction stoichiometry of consumers has been based on several assumptions along with the principal objective of not introducing any new parameters other than the control variables $\varepsilon_{C\left\{i\right\}}$ and $\Omega_{\chi \left\{j\right\},C\left\{i\right\}}$.

### 2.3. Reaction Kinetics

Two types of kinetic expressions govern reaction rates in the model, one for electromagnetic energy and another for chemical energy, which were developed in Vallino and Huber [17], but are briefly describe here. Phototrophs incorporate both types of kinetics, as the first reaction ($R_{1,P\left\{i\right\}}$, Equation (3)) utilizes electromagnetic radiation to drive $C_{P\left\{i\right\}}$ synthesis, while the chemical oxidation of $C_{P\left\{i\right\}}$ is used for ${𝕊}_{P\left\{i\right\}}$ synthesis in $R_{2,P\left\{i\right\}}$ (Equation (4)). Photosynthetic radiation enters a system on an areal basis, which differs from chemical energy that is volume based. Chemical reaction rates depend on the state of the system—reactant concentrations—while photoreaction rates depend on the rate of areal photon input; consequently, photosynthetic reaction rates are proportional to $I\left(t,z\right)⟦mmol-\gamma \;m^{-2}d^{-1}⟧$, which is the light intensity at time *t* and water depth z.

The rate, $\Delta I_{P\left\{i\right\}}\;$, at which high frequency photons, $\gamma_{H}$, are captured by phytoplankton photosynthetic machinery per unit volume $⟦mmol-\gamma \;m^{-3}d^{-1}⟧$, is given by,

(5) $$\Delta I_{P\left\{i\right\}}=k_{Chl}\Omega_{1,P\left\{i\right\}}\left[{𝕊}_{P\left\{i\right\}}\right]I{\left(t\right)}_{\zeta_{d}}$$

where $k_{Chl}⟦m^{2}\;{\left(mmol-C\right)}^{-1}⟧$ is the light attenuation coefficient by chlorophyll a [42], $\Omega_{1,P\left\{i\right\}}\left[{𝕊}_{P\left\{i\right\}}\right]$ is the fraction of phytoplankton biomass allocated to photosynthesis, and $I{\left(t\right)}_{\zeta_{d}}$ is the depth-averaged light intensity $⟦mmol-\gamma \;m^{-2}d^{-1}⟧$ for a well-mixed column of water of depth $\zeta_{d}⟦m⟧$ (Equation (S9)). Note for simplicity, we only consider blue light of 440 nm wavelength, as a full spectrum model would require considerably more development and is beyond the scope of this initial study.

As described above for reaction $R_{1,P\left\{i\right\}}$, $n_{1,P\left\{i\right\}}$ is the mmoles of photons needed to fix one mmole of CO_2_ under thermodynamic reversibility (see Equation (S6)), so the maximum rate of reaction $R_{1,P\left\{i\right\}}$ is given by $\frac{\Delta I_{P\left\{i\right\}}}{n_{1,P\left\{i\right\}}}$. However, the reaction rate can also be limited by CO_2_ plus HCO_3_^-^ concentration, so the overall rate expression for photon driven reaction, $R_{1,P\left\{i\right\}}$, is given by,

(6) $$r_{1,P\left\{i\right\}}=\frac{\Delta I_{P\left\{i\right\}}}{n_{1,P\left\{i\right\}}}F_{K}\left(c,\Lambda_{1,P},\varepsilon_{P\left\{i\right\}}\right)F_{T}\left(\Delta_{r}G_{1,P\left\{i\right\}},n_{1,P\left\{i\right\}}^{e}\right),$$

where $F_{K}$ is the kinetic drive [43] (described below) and $F_{T}$ is a thermodynamic drive as described by LaRowe et al. [44], which depends on the Gibbs free energy of reaction and the number of electrons transferred, $n_{1,P\left\{i\right\}}^{e}$, in the catabolic reaction, as given by (also see [17]),

(7) $$F_{T}\left(\Delta_{r}G_{j,\chi \left\{i\right\}},n_{j,\chi \left\{i\right\}}^{e}\right)=\frac{1}{1+\exp\left(\frac{\Delta_{r}G_{j,\chi \left\{i\right\}}/n_{j,\chi \left\{i\right\}}^{e}+F\Delta \psi }{RT}\right)},$$

where F is the Faraday constant and Δψ is the membrane potential set to 0.1 V for all simulations. Simply stated, as the reaction free energy goes to zero, $F_{T}$ drives the reaction rate to zero regardless of how favorable the kinetic drive [44,45].

The second class of reaction kinetics used for chemotrophs (i.e., bacteria) and consumers, as well as for the phytoplankton biosynthesis reaction, $R_{2,P\left\{i\right\}}$, is an adaptive Monod Equation [29], which has the general form given by,

(8) $$r_{j,\chi \left\{i\right\}}=\nu^{*}\varepsilon_{\chi \left\{i\right\}}^{2}\Omega_{j,\chi \left\{i\right\}}\left[{𝕊}_{\chi \left\{i\right\}}\right]F_{K}\left(c,\Lambda_{j,\chi },\varepsilon_{\chi \left\{i\right\}}\right)F_{T}\left(\Delta_{r}G_{j,\chi \left\{i\right\}},n_{j,\chi \left\{i\right\}}^{e}\right),$$

where the kinetic drive, $F_{K}\left(c,\Lambda_{j,\chi },\varepsilon_{\chi \left\{i\right\}}\right)$, is given by,

(9) $$F_{K}\left(c,\Lambda_{j,\chi },\varepsilon_{\chi \left\{i\right\}}\right)=\overset{n_{S}}{\underset{k=1}{\prod }}{\left(\frac{c_{k}}{c_{k}+\kappa^{*}\varepsilon_{\chi \left\{i\right\}}^{4}}\right)}^{\Lambda_{j,k,\chi }}.$$

The kinetic drive, $F_{K}$, depends on the concentrations, $c_{k}$ $⟦mmol\;m^{-3}⟧$, of the state variables of which there are $n_{S}$, and $\Lambda_{j,\chi }$ is a binary matrix that specifies the reactants used in reaction j for biological structure χ, which can be P, B, or C, where $\Lambda_{j,k,\chi }$ equals 1 if reactant $c_{k}$ is a reactant in reaction $R_{j,\chi }$; otherwise, $\Lambda_{j,k,\chi }$ equals 0. The maximum biomass-specific reaction rate, $\nu^{*}\varepsilon_{\chi \left\{i\right\}}^{2}⟦d^{-1}⟧$, in Equation (8) and the half saturation constant, $\kappa^{*}\varepsilon_{\chi \left\{i\right\}}^{4}⟦mmol\;m^{-3}⟧$, in the kinetic drive, Equation (9), are both parameterized by $\varepsilon_{\chi \left\{i\right\}}$. The fixed parameters $\nu^{*}$ and $\kappa^{*}$ have been chosen so that as $\varepsilon_{\chi \left\{i\right\}}$ varies from 0 to 1, the growth kinetics describe a family of curves that represent the growth rate of oligotrophs to copiotrophs, respectively [29]. Since $\nu^{*}$ and $\kappa^{*}$ are held constant at 350 d^−1^ and 5000 mmol m^−3^, respectively, for all functional groups and reactions, except detritus decomposition, there are no adjustable parameters other than the two control variables $\varepsilon_{\chi \left\{i\right\}}$ and $\Omega_{j,\chi \left\{i\right\}}$ governing reaction rates and stoichiometry. For decomposition of detrital organic matter given by reactions $R_{2,B\left\{i\right\}}$ and $R_{3,B\left\{i\right\}}$, $\nu_{D}^{*}$ replaces $\nu^{*}$ to reflect the slower kinetics associated with detritus utilization, where $\nu_{D}^{*}$ is set to 175 d^−1^.

Equations (8) and (9) also govern consumer, ${𝕊}_{C\left\{i\right\}}$, reaction rates (see Equation (S58)), but the number of reactions is the total number of prey in the simulation $\left(n_{P}+n_{B}+n_{C}\right)$, so that the resource allocation variable, $\Omega_{\chi \left\{j\right\},C\left\{i\right\}}$, is a matrix of traits with size $(n_{P}+n_{B}+n_{C})\times n_{C}$. This matrix grows rapidly as the number of ecotypes in the model are increased, which introduces some challenges in exploring trait space as discussed in Section 2.5 below. The details for each reaction are provided in the Supplementary Material.

### 2.4. Model Domain and Simulation Details

For simplicity, the model domain is a pond-like cylindrical reservoir of depth $\zeta_{d}$ and surface area A that is illuminated at the surface with both diel and seasonal solar radiation and can exchange O_2_ and CO_2_ with the atmosphere. The pond is modeled as a well-mixed system (0D) with equal input and output flows, $F⟦m^{3}d^{-1}⟧$, and a fixed volume, $V⟦m^{3}⟧$, which defines a dilution rate given by $D=\frac{F}{V}⟦d^{-1}⟧$. A governing set of ordinary differential equations (ODEs, Equation (S1)) is derived from mass balances (Supplementary Materials S2.3) around the six constituents $\left(H_{2}CO_{3},\;O_{2},\;NH_{3},\;C_{L},\;C_{D},\;N_{D}\right)$ and $n_{P}$ phytoplankton, $n_{P}$ phytoplankton carbon stores, $n_{B}$ bacteria, and $n_{C}$ consumers. An additional three ODEs (Equations (S70)–(S72)) integrate irreversible entropy production, $\dot{\sigma }$, to obtain total entropy production, $\sigma^{T}$, with contributions from reactions, $\sigma^{R}$, particles, $\sigma^{P}$, and water, $\sigma^{W}$. All simulations are run for two years with constant inputs at a dilution rate of 0.2 d^−1^ unless otherwise specified.

### 2.5. Optimize Trait-Based Model

In previous work, we have used optimal control to determine how the growth kinetics of each functional group changes over both time and space to maximize σ [17,25,26,29]. In that approach, only a single state variable is used to represent each functional group, but the growth characteristics of each group can change over space and time as dictated by the control variables. A disadvantage of the approach is that the dimension of the optimal control problem grows rapidly with each spatial dimension added and becomes computationally prohibitive for 2D and 3D spaces, at least under the current numerical approach. Even though the model developed here is 0D, we explore a new approach that uses trait-based modeling that can be extended to 2D and 3D environments.

In trait-based modeling [46], growth parameters are considered as traits, reflecting optimal growth conditions, such as temperature and light intensity, that incorporate appropriate tradeoffs, such as specific growth is negatively correlated with substrate affinity. The model domain is populated with many ecotypes in each functional group and trait values are randomly selected from an appropriate distribution, so that models start with high in silico diversity. The models can have hundreds of state variables capturing diverse ecotypes, but as model simulations proceed, in silico natural selection culls ecotypes from the population that have poor growth kinetics for the local environmental conditions. Furthermore, by continuously seeding the simulation with low concentrations of all ecotypes, if the simulated environment changes, new ecotypes can be selected for, which removes some of the problems that plague classic biogeochemistry models regarding population restructuring and the need to recalibrate parameters. To achieve good numerical coverage for a particular trait, such as substrate affinity, a sufficient number of ecotypes with that trait must be included in the simulation, so a large number of traits can lead to a computationally prohibitive number of state variables, which is particularly true when consumers are included in the network.

As mentioned above, the consumer trait matrix $\Omega_{\chi \left\{j\right\},C\left\{i\right\}}\in {\mathbb{R}}^{(n_{P}+n_{B}+n_{C})\times n_{C}}$ determines prey preferences for consumer ${𝕊}_{C\left\{i\right\}}$, but it also determines the trophic structure of the food web, because consumer C{i} can consume consumer C{j}, which in turn could consume another consumer to produce a four-level trophic food web. More importantly, as the number of phytoplankton, bacteria, and consumer ecotypes are added to the model to explore trait space, the size of $\Omega_{\chi \left\{j\right\},C\left\{i\right\}}$ increases roughly as the square of the number of prey. For instance, if 10 ecotypes are used for P and B, then a single consumer will have 21 traits (row dimension of $\Omega_{\chi \left\{j\right\},C\left\{i\right\}}$), which would need to be explored by adding more consumers with different values for the 21 traits, but adding more consumers increases the column dimension of $\Omega_{\chi \left\{j\right\},C\left\{i\right\}}$ and the number of traits used by each consumer. Consequently, in this configuration, the traits space for ${𝕊}_{C\left\{i\right\}}$ increases faster than it can be explored. One solution is to limit the number of prey that a consumer can target, but this places strong constraints on the structure of the food web, which is something we wanted to avoid in this study. This conundrum lead to the development of a new optimal trait-based modeling approach. Instead of exploring trait space by adding many ecotypes to a model and relying on in silico selection to find the best trait values, we used a hybrid approach between trait-based modeling and our previous optimal control modeling. In this new approach, we populate the model with relatively few ecotypes for each functional group, then use optimization to search for trait values that maximize entropy production over a specified time interval instead of randomly assigning trait values.

All simulations were run with a fixed depth of 1 m, unless otherwise noted, because a deeper water column would require use of a 1D transport model so that local entropy production could be optimized at each depth. While dissolved constituents are assumed to be well mixed, this does not apply to light, which exhibits an exponential decrease in intensity as a function of depth and the concentration of particles and Chl a. As discussed in Vallino and Huber [17], entropy production associated with dissipation of electromagnetic energy, or any energy potential that is quickly dissipated abiotically, must be maximized locally instead of globally to obtain solutions that are consistent with biology. This can be achieved by discretizing the water column into thin, ~ 1 m, layers, then conducting EP optimization in each layer. Consequently, simulations presented here are effectively one layer at the surface.

### 2.6. Temporal Strategies

Three phytoplankton temporal strategies are investigated here that affect how phytoplankton biomass is allocated to the two associated reactions, $R_{1,P\left\{i\right\}}$ and $R_{2,P\left\{i\right\}}$ (Table 1): (1) Balanced growth; (2) passive C storage; (3) circadian resource allocation. For the first case that lacks temporal strategy, it is assumed that phytoplankton are limited to balanced growth, so that the ratio of ${𝕊}_{P\left\{i\right\}}$ to $C_{P\left\{i\right\}}$, or C:N ratio of phytoplankton, remains constant during the simulation, which is achieved by constraining reaction rates $r_{1,P\left\{i\right\}}$ and $r_{2,P\left\{i\right\}}$ as given by Equations (S80)–(S82) and described in Supplementary Materials S3.2. For passive C storage, $r_{1,P\left\{i\right\}}$ and $r_{2,P\left\{i\right\}}$ are not coupled, which allows $C_{P\left\{i\right\}}$ to increase and decrease relative to ${𝕊}_{P\left\{i\right\}}$ in a manner reminiscent of Droop’s formulation [47]. However, in passive storage, there is no temporal variation in resource allocation, so $\Omega_{1,P\left\{i\right\}}$ (allocation to CO_2_ fixation reaction, $R_{1,P\left\{i\right\}}$) and $\Omega_{2,P\left\{i\right\}}$ (allocation to phytoplankton biosynthesis reaction, $R_{2,P\left\{i\right\}}$), remain constant for the duration of the simulation. In circadian allocation, the resource allocation trait, $\Omega_{1,P\left\{i\right\}}$, can vary with time. Initial studies used a sinusoid function for $\Omega_{1,P\left\{i\right\}}\left(t\right)$, Equation (S75), where frequency, $f_{P\left\{i\right\}}$, and phase, $\varphi_{P\left\{i\right\}}$, were used as traits and determined by EP maximization along with all other traits. While this approach worked, the global optimum was always found with $f_{P\left\{i\right\}}=1\;d^{-1}$, but this global solution was computationally difficult to locate due to the narrowness of the optimum (Figure S1). To increase computational speed, we choose a square-wave function for $\Omega_{1,P\left\{i\right\}}\left(t\right)$, Equation (S76), that varies on a diel cycle, where three trait parameters, $t_{On\left\{i\right\}}$, $t_{Off\left\{i\right\}}$, and $\Omega_{amp\left\{i\right\}}$ are used to set the time of step-up, step-down, and amplitude of the square wave, respectively. Setting $t_{On\left\{i\right\}}$ and $t_{Off\left\{i\right\}}$ to 0 and 1 d^−1^, respectively, produces the same results as the passive storage strategy.

### 2.7. Optimization and Computational Approach

As discussed in Section S3, all simulations were conducted on a small five-node computer cluster (90 CPUs), where each CPU solved a local optimization problem from an initial location in trait space that was selected from sampling a Latin hypercube to facilitate locating the global maximum. The basic algorithm is as follows. The optimization routine (hyperBOB) maximizes total entropy production, $\sigma^{T}$, by setting the values of the trait variables ($\varepsilon_{\chi \left\{i\right\}}$, $\Omega_{j,\chi \left\{i\right\}},\;t_{On\left\{i\right\}},\;t_{Off\left\{i\right\}}\;\mathrm{and}\;\Omega_{amp\left\{i\right\}}$) and passing them to the ODE integrator (BiM [48]), which determines how state variables and entropy production vary over a two year period. Total entropy produced over the two-year period is returned to the optimization routine that then adjusts the values of the trait variables to maximize entropy production based on a quadratic reconstruction of the optimum surface, which obviates the need for the gradient of $\sigma^{T}$ (see BOBYQA [49]). Iteration ends once the search region decreases to a user specified minimum radius for each processor.

Most of the simulations run in this study use a 1P1B1C food web model, but we also explore other small networks. In addition to model runs that explored the three temporal strategies described in Section 2.6 and Supplementary Materials Section S3.2, we also examine how $\sigma^{T}$ changes with increasing food web complexity as well as changes in chemical versus electromagnetic energy input.

## 3. Results

We investigate three aspects of the MEP-optimized trait-based model described above, which include (1) how the three phytoplankton temporal strategies alter the food webs ability to dissipate electromagnetic and chemical free energy, (2) how food web complexity changes entropy production, and (3) how the food web adjusts to changes in the relative inputs of electromagnetic versus chemical free energy. All simulations are run for two years that include both diel and seasonal changes in solar input but constant inputs of chemical constituents at the specified dilution rate. Only simple food webs are explored in this study, consisting of one phytoplankton, one bacterium, and one consumer, 1P1B1C, and two other configurations consisting of 2P2B2C and 3P3B3C.

### 3.1. Phytoplankton Temporal Strategies and Entropy Production

As discussed in Section 2.6 and Section S3.2, the three phytoplankton temporal strategies we explore here consist of a balanced growth strategy with fixed phytoplankton stoichiometry, a passive strategy where phytoplankton can store carbon fixed from photosynthesis for later use, and an explicit clock-based, or circadian, strategy for resource allocation between carbon fixation, $R_{1,P\left\{i\right\}}$, and phytoplankton biosynthesis, $R_{2,P\left\{i\right\}}$, reactions on a diel cycle. However, before presenting the results from those simulations, it is useful to characterize the magnitude of energy inputs for the nominal simulations as well as examine entropy production and phytoplankton dynamics for randomly selected trait values.

The nominal simulations examine a 1P1B1C food web model operated at a dilution rate of 0.2 d^−1^ with input concentrations given in Table 2. Under these input conditions, aerobic oxidation of all the supplied organic carbon $\left(C_{L}+C_{D}\right)$ in a 1 m deep pond with 1 m^2^ surface over a two-year period produces 0.025 MJ K^−1^ of entropy from chemical energy dissipation, while the dissipation of solar radiation input from a latitude of 42° over the 1 m^2^ surface produces 27.1 MJ K^−1^ of entropy over the same two year period. Consequently, energy input and entropy production from electromagnetic radiation is more than 1000 times greater than that from chemical free energy in the nominal simulations. In Section 3.3, results from simulations where energy inputs are similar will be examined, but in this section and the next, nominal conditions (Table 2) will be used that vastly favor dissipation of electromagnetic free energy.

**Figure 2:**
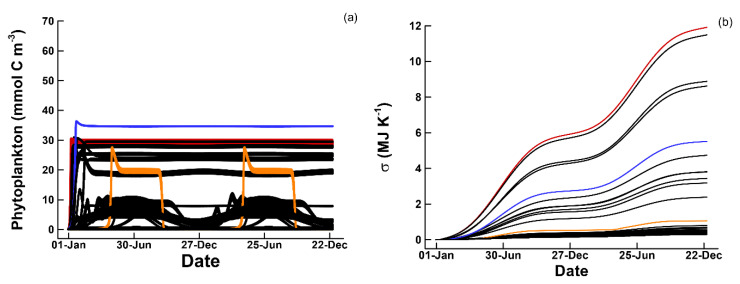
(**a**) Phytoplankton concentration and (**b**) cumulative total entropy production, $\sigma^{T}$, over two years for 90 simulations with random selection of trait values for a 1P1B1C food web configuration using nominal input concentrations (Table 2). Three of the 90 solutions are highlighted by color in (**a**) and (**b**) corresponding to high (red), intermediate (blue), and low (orange) entropy production.

The number of traits, $n_{T}$, that need to be set depends on the size of the food web and is given by, $4n_{P}+3n_{B}+\left(1+n_{P}+n_{B}+n_{C}\right)n_{C}$; consequently, the 1P1B1C has an 11 dimensional trait space. To examine entropy production and food web dynamics when traits are just randomly assigned, we conducted 90 simulations where the trait values were randomly selected based on Latin hypercube sampling. In these simulations, total entropy production, $\sigma^{T}$, over a two-year period varied from 0.2994 to 11.91 MJ K^−1^, and phytoplankton reached a maximum concentration of approximately 35 mmol m^−3^ with various diel and seasonal dynamics (Figure 2). The random solutions serve as a null model that the optimum solutions can be compared to with respect to entropy production. They also reveal a complex relationship between phytoplankton dynamics (Figure 2a) and entropy production (Figure 2b). For instance, the solution with the highest phytoplankton concentration only produces entropy at an intermediate level (Figure 2, blue lines). Furthermore, if the growth efficiencies for phytoplankton, bacteria, and consumers are set to zero so that no growth occurs, the total entropy produced is 0.3077 MJ K^−1^ due to particle absorption of solar radiation by biomass in the input (Table 2).

**Figure 3:**
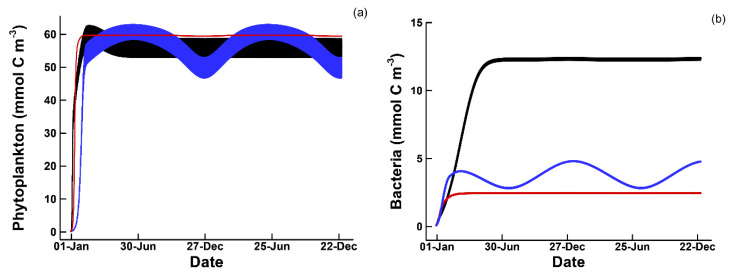

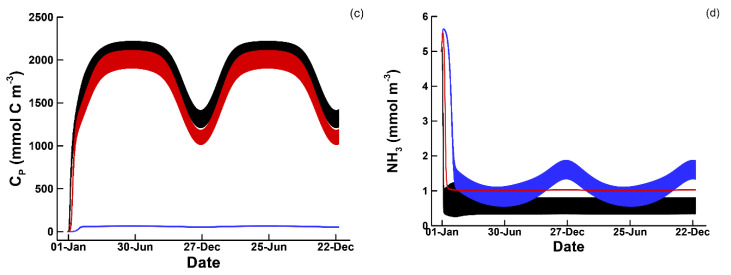
Variations in (**a**) phytoplankton, (**b**), bacteria, (**c**) phytoplankton carbon storage $C_{P}$, and (**d**) ammonium concentrations (mmol m^−3^) over the two-year simulations under the three different temporal strategies: Blue, balanced growth; red, passive storage; black, circadian allocation.

For each temporal strategy, optimizations were run starting from 90 different initial locations within trait space that were selected by Latin hypercube sampling (see Section S3) to increase the likelihood of locating the global optimum. All three temporal strategies produced similar phytoplankton concentrations between 50 and 60 mmol m^−3^ (Figure 3a) and all reduced ammonium concentrations from the input of 5 mmol m^−3^ to 0.5 to 2 mmol m^−3^ (Figure 3d). Similarly, detrital nitrogen, $N_{D}$, was drawn down from 7 mmol m^−3^ to approximately 1.5, 1.2, and 0.7 mmol m^−3^ in the balanced growth, passive, and circadian simulations, respectively (not shown). In all three simulations, optimal solutions excluded consumers from growing by selecting consumer growth efficiencies, $\varepsilon_{C\left\{1\right\}}$, near 0 or 1 (Table 3). Bacteria concentrations were highest in the circadian strategy, at 12 mmol m^−3^, and lowest in the passive strategy, at 2.5 mmol m^−3^ (Figure 3b). Both the circadian and passive strategies accumulated high concentrations of phytoplankton internal carbon, $C_{P\left\{1\right\}}$, with a strong seasonal signal, while $C_{P\left\{1\right\}}$ in the balanced growth strategy never exceeded 70 mmol m^−3^ (Figure 3c). The high $C_{P\left\{1\right\}}$ concentrations in the passive and circadian strategies produced phytoplankton C:N ratios that varied from 115 to 230, and from 140 to 270, respectively, while the phytoplankton C:N ratio in the balanced growth solution was held fixed at 14 (data not shown).

As defined in Section S2.4 of the Supplemental Materials, total entropy production is summed over three different sources of entropy production that include photon energy absorbed and dissipated by water, $\sigma^{W}$, and particles, $\sigma^{P}$, and dissipation of chemical free energy by reactions, $\sigma^{R}$. Dissipation of photon free energy by water is self-explanatory, and dissipation by nonphotoreactive particles, such as bacteria and consumers, is similar to dissipation by water, except photon absorption depends on particle concentration, but otherwise contributes directly to $\sigma^{P}$. In both cases, photon energy just warms the surrounding water. Free energy released by chemical reactions contributes directly to $\sigma^{R}$ as defined by Equation (1). However, photon free energy capture by phytoplankton photosynthetic machinery, such a Chl a and accessory pigments represented by $\Omega_{1,P\left\{i\right\}}\left[{𝕊}_{P\left\{i\right\}}\right]$ in the model, contributes to both $\sigma^{R}$ and $\sigma^{P}$. If the CO_2_ fixation reaction rate, $r_{1,P\left\{i\right\}}$, is not constrained by either the thermodynamic, $F_{T}$, or kinetic, $F_{K}$, drivers, then the free energy released by $R_{1,P\left\{i\right\}}$ contributes entirely to $\sigma^{R}$. However, if $r_{1,P\left\{i\right\}}$ is reduced by $F_{T}$ (which occurs as $\varepsilon_{P\left\{i\right\}}$ approaches 1) or by $F_{K}$ (which occurs as the concentration of CO_2_ + HCO_3_^−^ becomes low relative to $\kappa^{*}\varepsilon_{P\left\{i\right\}}^{4}$) then some of the photon free energy dissipated contributes to $\sigma^{P}$ as defined by Equations (S13)–(S15), because the phytoplankton photosynthetic machinery is behaving like a particle then. This accounting does not change the model simulation results, just how entropy production results are tabulated.

In terms of entropy production over the two-year simulation, the circadian strategy produced the greatest amount at 19.74 MJ K^−1^, followed closely by the passive strategy at 18.95 MJ K^−1^, which were both much higher than the balanced growth solution, which only produced 3.984 MJ K^−1^ (Table 3). The source of entropy production for all three strategies is largely due to light attenuation due to particles in the water column, with $\sigma^{P}$ accounting for 86%, 94%, and 94% for the balanced growth, passive storage, and circadian strategies, while entropy production from reactions, $\sigma^{R}$, only accounts for 7.1%, 5.2%, and 5.0% of $\sigma^{T}$, respectively (Table 3). These results are not surprising given the amount of free energy entering the system from light versus chemical potential. For aquatic systems, dissipating electromagnetic energy is mostly about synthesizing particles to intercept high frequency photons and dissipate their energy as heat [17]. Light attenuation by water, $\sigma^{W}$, contributes 6.9%, 0.89%, and 0.82% to entropy production for balanced, passive, and circadian strategies, respectively.

**Figure 4:**
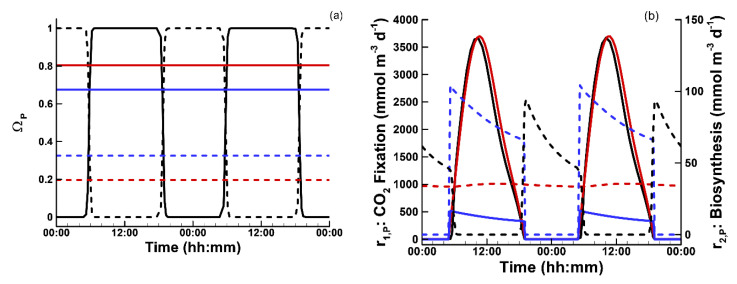
(**a**) How phytoplanton resource allocation, $\Omega_{1,P\left\{1\right\}}$ (solid lines) and $\Omega_{2,P\left\{1\right\}}$ (dashed lines) and (**b**) reactions for CO_2_ fixation, $r_{1,P\left\{1\right\}}$, (solid lines) and biosynthesis, $r_{2,P\left\{1\right\}}$ (dashed lines), vary over a two-day period in the two-year simulations associated with balanced growth (blue lines), passive storage (red lines), and circadian resource allocation (black lines).

The entropy production differences between the three strategies can be understood by considering how phytoplankton biomass is allocated to the carbon fixation reaction, $R_{1,P\left\{1\right\}}$, and the biosynthesis reaction, $R_{2,P\left\{1\right\}}$, that is determined by $\Omega_{1,P\left\{1\right\}}$, which in turn depends on the three trait values, $t_{On\left\{1\right\}}$, $t_{Off\left\{1\right\}}$, and $\Omega_{amp\left\{1\right\}}$, that govern the nature of the square wave function, Equation (S77). For both the balanced growth and passive storage strategies, $t_{On\left\{1\right\}}$ and $t_{Off\left\{1\right\}}$ are constrained to be 0 and 1, respectively, so that $\Omega_{1,P\left\{1\right\}}$ remains constant at the value given by $\Omega_{amp\left\{1\right\}}$, while $t_{On\left\{1\right\}}$ and $t_{Off\left\{1\right\}}$ can vary over the range [0,1] for the circadian strategy, which produces a diel square wave when either $t_{On\left\{1\right\}}>0$ or $t_{Off\left\{1\right\}}<1$ (Figure 4a). In the optimal circadian strategy, all phytoplankton resources are allocated to CO_2_ fixation (Table 3; $\Omega_{amp\left\{1\right\}}=1$) when the fractional time of day, $t_{D}$, falls within the interval $0.2389\;d\leq t_{D}\leq 0.7799\;d$, and redirected to biosynthesis outside the interval (Figure 4a, black lines). In both the balanced growth and passive storage strategies, the optimal solutions locate a compromise between allocation of biomass to $R_{1,P\left\{1\right\}}$ versus $R_{2,P\left\{1\right\}}$, where 67.5% and 80.4% of biomass is allocated to carbon fixation, $R_{1,P\left\{1\right\}}$, at all times for the balanced growth and passive storage optimal solutions, respectively ($\Omega_{amp\left\{1\right\}}$, Table 3; Figure 4a, blue and red lines). These different allocation strategies significantly impact the rates of the two reactions associated with phytoplankton ($r_{1,P\left\{1\right\}}$ and $r_{2,P\left\{1\right\}}$).

In the balanced growth strategy, $r_{1,P\left\{1\right\}}$ and $r_{2,P\left\{1\right\}}$ must be coupled by definition of balanced growth; consequently, growth can only occur during the day, and any limitations on growth during daylight, such as due to NH_3_ available, also limits CO_2_ fixation rate. For instance, considering just two days in the two year simulation (Figure 4b), $r_{1,P\left\{1\right\}}$ and $r_{2,P\left\{1\right\}}$ in the balanced growth strategy (Figure 4b, blue lines), both equal 0 at night, and the decrease in both CO_2_ fixation (blue solid line) and biosynthesis (blue dashed line) during the day is due to NH_3_ limitation occurring (Figure 4b). The passive storage strategy avoids the reaction coupling limitation, so that biosynthesis can occur at night (Figure 4b, dashed red line), but because resource allocation is fixed in the passive strategy, all cellular resources cannot be allocated to growth at night, nor CO_2_ fixation in the day. The circadian strategy relaxes this problem by allocating resources dynamically, so that growth at night can be maximized (Figure 4b, black dashed line), yet still be able to fix CO_2_ during daylight at maximum rate as well (Figure 4b, black solid line). However, since free energy dissipated by chemical reactions contribute little to entropy production in all strategies (Table 3, $\sigma^{R}$), the differences in total entropy production lies elsewhere.

The much higher entropy production by the passive and circadian strategies over the balanced growth strategy is due to the much higher concentration of light absorbing particles. While phytoplankton concentrations are similar in all three strategies (Figure 3a), phytoplankton carbon storage, $C_{P\left\{1\right\}}$, that contributes to light attenuation (Equation (S8)) is much higher in the passive and circadian strategies (Figure 3c). In order to store carbon, phytoplankton must grow in size, which increases their cross-sectional area for light interception. The intercepted light cannot be used for photosynthesis by definition, but the electromagnetic energy $C_{P\left\{1\right\}}$ absorbs is converted to heat that results in entropy production. Under the nominal conditions (Table 2), the planktonic community is N limited, so biomass, S, accumulation is constrained; however, $C_{P\left\{1\right\}}$ does not contain N and is not constrained by N availability. Both the passive and circadian strategies increase entropy production by investing in $C_{P\left\{1\right\}}$ synthesis using electromagnetic energy, and the circadian strategy does this slightly more effectively due to temporal control on resource allocation. Not surprisingly, entropy production in the balanced growth solution is increased by increasing either $NH_{3}$ or $N_{D}$ concentrations in the feed. For instance, at an $N_{D}$ input concentration of 50 mmol m^−3^, the balanced growth solution increases $\sigma^{T}$ to 15.33 MJ K^−1^ and maintains a phytoplankton concentration of ~300 mmol m^−3^.

### 3.2. Entropy Production and Food Web Complexity

Here we examine the effect of increasing food web complexity by adding more ecotypes to each functional group. In particular, we compare the solutions from the 1P1B1C configuration discussed above to 2P2B2C and 3P3B3C configurations run under the nominal input conditions (Table 2). These simulations, which were also run at a dilution rate of 0.2 d^−1^, produced nearly the same amount of entropy as the 1P1B1C solution (Table 4), and nutrient and organism dynamics were very similar to the 1P1B1C solutions as well, with only minor or duplicate contributions from the additional ecotypes (data not shown). For instance, in the 3P3B3C food web using passive storage strategy, two phytoplankton exhibited nearly identical dynamics and each attained a steady state concentration of ~30 mmol m^−3^, so when summed together they were equivalent to the 1P1B1C solution (Figure 3a, red line). As in the 1P1B1C solutions, consumers were nearly absent. The additional ecotypes in the more complex food web were effectively superfluous as far as the entropy maximization is concerned. However, the complexity of the food web became more important as dilution rate was increased, as well as the circadian strategy compared to the passive strategy.

When the three strategies along with the three different food webs were run under nominal input concentrations but at a dilution rate of 1.5 d^−1^, the added food web complexity and the circadian strategy showed enhanced entropy production relative to the other simulations (Table 5). There is approximately a 7% to 12% increase in $\sigma^{T}$ associated with the increase in food web complexity from 1P1B1C to 2P2B2C or from 2P2B2C to 3P3B3C regardless of the temporal strategy employed, but a much greater increase in $\sigma^{T}$ occurred as temporal strategies were changed. There is approximately a 320% increase in $\sigma^{T}$ as the strategy was changed from balanced growth to passive storage. In fact, phytoplankton in the solutions using the balanced growth strategy were near washout conditions at a dilution rate of 1.5 d^−1^, as their concentrations only attain ~1 mmol m^−3^ for a short period during the peak of summer. When the temporal strategy was switched from passive to circadian, there was approximately a 130% increase in $\sigma^{T}$, which indicates the usefulness of an explicit clock in improving entropy production over the passive solution. Furthermore, optimal solutions at high dilution rates exhibited complementary when more complex food webs were used.

**Figure 5:**
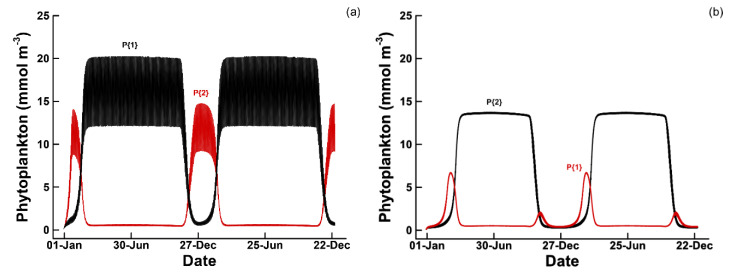
Phytoplankton concentration for a 2P2B2C food web configuration at a dilution rate of 1.5 d^−1^ using (**a**) the circadian allocation strategy versus (**b**) the passive storage strategy.

In general, most of the complex food web (2× and 3×) solutions did not show much complementarity between ecotypes at low dilution rates; however, when dilution rate was increased to 1.5 d^−1^ or more, phytoplankton (as well as the other functional groups to a lesser extent) exhibited complementary in solutions using the 2P2B2C or 3P3B3C food webs with either the passive storage or circadian strategies (Figure 5). For instance, at a dilution rate of 1.5 d^−1^, the best circadian solution selects for phytoplankton with traits that are complementary with respect to winter versus summer (Figure 5a, red versus black lines). Note, simulations did not investigate seasonal fluctuations in temperature, just solar radiation. In the circadian solution, $\varepsilon_{P\left\{1\right\}}$ and $\varepsilon_{P\left\{2\right\}}$ equal 0.341 and 0.401, respectively, which allows ${𝕊}_{P\left\{2\right\}}$ to grow slightly more efficiently than ${𝕊}_{P\left\{1\right\}}$ giving the former an advantage during winter when light intensity is lower and NH_3_ is ~2 mmol m^−3^ higher. The advantage of the circadian strategy over the passive strategy is evident in phytoplankton concentrations between the two simulations. At the same dilution rate of 1.5 d^−1^, the passive strategy has lower summer time phytoplankton concentration, (P{2}, Figure 5b, black line), and the winter ecotype, P{1}, is closer to being washed out of the system (Figure 5b, red line), which results in less entropy production compared to the circadian strategy (Table 5). At a dilution rate of 2.0 d^−1^, the 2P2B2C food web using the circadian strategy has an entropy production of 1.5042 MJ K^−1^ and looks very similar to Figure 5b, which implies the circadian strategy has approximately a 0.5 d^−1^ specific growth rate advantage over the passive strategy, which only produces 0.5943 MJ K^−1^ at the 2.0 d^−1^ dilution rate.

### 3.3. Dissipation of Chemical versus Electromagnetic Free Energy

As mentioned earlier, dissipation of all electromagnetic free energy produces more than 1000 times the entropy than dissipation of the chemical free energy under nominal inputs (Table 2). In this section, we examine simulations where the electromagnetic energy is reduced by a factor of 10 $\left(I_{0}^{M}=40,600.\;\left(mmol-\gamma \;m^{-2}\;d^{-1}\right)\right)$, and the chemical free energy input is increased by a factor of 100 by increasing the feed concentration of $C_{L}$ from 10 mmol m^−3^ to 12 mol m^−3^. With these changes, maximum possible entropy produced over a two-year period from electromagnetic radiation reduces to 2.7068 MJ K^−1^ and that from chemical free energy increases to 2.8061 MJ K^−1^. Simulations are run at a dilution rate of 0.2 d^−1^ with a 1P1B1C food web configuration using only the circadian temporal strategy, and we only consider a single optimization run using the standard 90 initial conditions in trait space based on Latin hypercube sampling.

**Figure 6:**
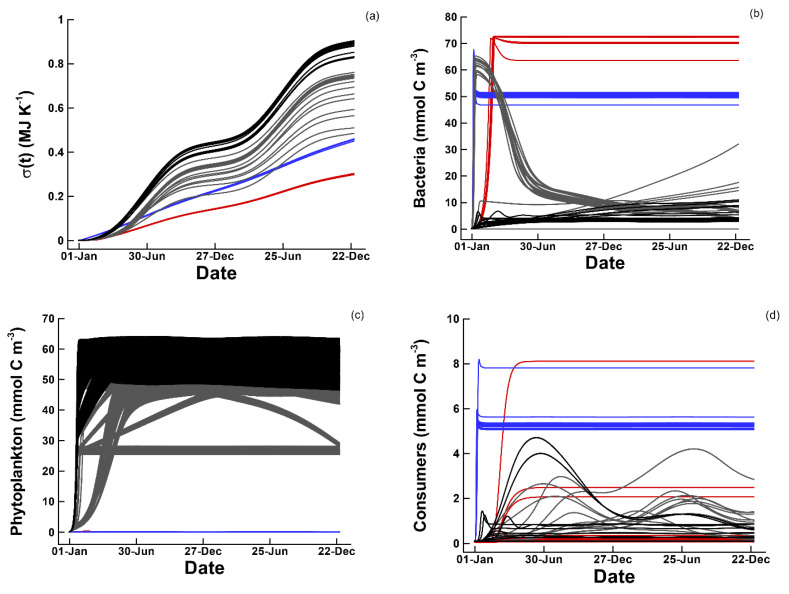
All 90 solutions from simulations using a 1P1B1C food web configuration with phytoplankton circadian allocation strategy where the input $C_{L}$ concentration has been increased to 12 mole m^−3^ and the solar radiation has been decreased by a factor of 10 so that electromagnetic and chemical free energy inputs are nearly equal. (**a**) Cumulative total entropy production, (**b**) bacteria, (**c**) phytoplankton, and (**d**) consumer concentrations over the two year simulation, where the line colors highlight solutions grouped around the three different optimum attractors corresponding to bacteria only (red), bacteria plus consumers (blue), and phytoplankton (grey and black).

Inspection of all 90 simulations reveals what appears to be three different types of solutions based on entropy production (Figure 6a). The first 36 solutions all produce nearly the same amount of entropy over the two-year period of 0.3019 MJ K^−1^ (Figure 6a, red lines), and all these solutions invest mainly in bacterial growth (Figure 6b, red lines) to oxidize $C_{L}$ from the initial 12.00 down to 11.232 mol m^−3^ but leave $C_{D}$ unused. No phytoplankton are produced (Figure 6c, red lines), and for most solutions, consumers remain at low concentrations (Figure 6d, red line). The next 16 solutions locate an entropy maximum that is a little higher at an average of 0.4583 MJ K^−1^ (Figure 6a, blue lines). These solutions do not invest in phytoplankton either and still produce entropy by bacterial oxidation of $C_{L}$, but these solutions lower the concentration of $C_{L}$ further to 10.485 mol m^−3^ by investing some N resources in consumers, which results in lower bacteria concentrations (Figure 6b,d, blue lines). By investing in consumers, which remineralize N in bacteria as NH_3_ and $N_{D}$ by grazing, the strategy reduces the N limitation on bacterial growth by rapid recycling of N that allows bacteria to consume and oxidize more $C_{L}$ and produce more entropy than solutions without consumers, even though bacteria biomass is lower, bacterial production is higher.

The last 38 solutions instead invest in phytoplankton (Figure 6c, grey and black lines) to exploit the electromagnetic free energy, producing the greatest amount of entropy at 0.9041 MJ K^−1^ and imparting smooth oscillations in cumulative entropy production due to the seasonal nature of solar radiation over the two year period (Figure 6a, grey and black lines). There appears to be either several local optimum in these solutions, or the optimization routine may have had difficulty locating the true global optima because the entropy production from the 38 solutions span a range from a minimum of 0.4846 to the maximum of 0.9041 MJ K^−1^ (Figure 6a, grey and black lines). These solutions invest minimally in bacteria (Figure 6b), which are used primarily to remineralize $N_{D}$ to NH_3_, which is evident in values of $\Omega_{1,B\left\{1\right\}}$, $\Omega_{2,B\left\{1\right\}}$, and $\Omega_{3,B\left\{1\right\}}$ traits. In the first 52 of 90 solutions discussed above, the reaction for bacterial growth, $R_{1,B\left\{1\right\}}$, is heavily favored with $\Omega_{1,B\left\{1\right\}}≅0.95$, with the remainder of the bacterial catalyst allocated to $N_{D}$ decomposition by $R_{3,B\left\{1\right\}}$ with $\Omega_{3,B\left\{1\right\}}$ set to 0.05. In the phytoplankton-based strategy, the weighting of bacterial catalyst to reactions is more variable, but solutions are in the neighborhood defined by $\Omega_{1,B\left\{1\right\}}≅0.5$ and $\Omega_{3,B\left\{1\right\}}≅0.5$. In all solutions, $\Omega_{2,B\left\{1\right\}}≅0$, so that $C_{D}$ remains unused.

## 4. Discussion

One of the challenges in modeling biogeochemistry is that numerous parameters are needed to describe growth kinetics and predator–prey interactions of the organisms that comprise a microbial food web [50]. Because natural microbiomes consist of hundreds to thousands of species whose growth kinetics and interactions are poorly known, models typical aggregate organisms into functional groups, such as the three used in this study. Even after aggregation, many dozens of parameters remain, and their values are unknown and only crudely bounded. Consequently, parameter values are “tuned” so that the sum of the squared residuals between model output and observations is minimized [51,52,53]. Because the models contain little to no fundamental principles other than conservation of mass, the standard approach becomes a non-linear modeling fitting exercise, and there is often multiple parameter values that can fit the limited observations equally well [51]. While the resulting models do interpolate observations well, their ability to predict beyond the observations used for calibration is very limited, because fundamental information on how microbial communities organize and function is lacking in their development. Furthermore, the models require recalibration as environmental conditions change even for interpolation, because new conditions drive species succession that have different growth characteristics.

To address some of the deficiencies of classic food web models, there is a long history of applying thermodynamic approaches to understand ecosystem function that forego predicting fine-scale details, such as the dynamics of hundreds of species, to improve long-term prediction fidelity, which has analogy to predicting climate rather than weather [5,54,55]. More recently, trait-based modeling has been developed that avoids the brittleness of classic models by retaining high species diversity that allows in silico community succession. The current formulation of the thermodynamically based MEP model gains inspiration from trait-based models [46] that have advanced to include size-structured food webs [56] as well as functional gene expression [57] and adaptation [58]. However, we have proposed a new approach where traits are not randomly assigned but rather are determined from maximizing entropy production, which allows fewer ecotypes to be simulated thereby reducing model state dimension. While the MEP trait-based model does require a computationally costly optimization, once trait values are determined, the model can be run without optimization as we will discuss at the end of this section.

The model presented in this manuscript is designed under the hypothesis that systems organize to dissipate free energy, and specifically that living systems maximize entropy production over a characteristic time scale while abiotic systems maximize entropy production instantaneously. Interestingly, the objective function is not growth oriented, as that alone does not dissipate free energy, but rather to produce catalysts that dissipate chemical free energy or produce particles that intercept light and dissipate it as heat. Unlike most bio-centric models that strive to grow organisms and/or maximize growth rate, the emphasis here is on free energy dissipation and how the system can organize to maximize its rate of destruction. The basic optimization objective is to synthesize just enough catalyst given available resources to maximize the dissipation of free energy. As Lineweaver and Egan [59] noted, ‘This represents a paradigm shift from “we eat food” to “food has produced us to eat it”.’ Furthermore, we have placed emphasis on removing biological growth parameters and replacing them with control variables, or traits, which are dynamically adjusted between model runs to maximize EP over a specified time interval. If the model does not produce realistic results or compares poorly to observations, it indicates that either model structural errors exist or that the MEP hypothesis is falsified for biogeochemistry [23]. The MEP approach also permits quantitative comparison of different energy sources to a system, such as our comparison of chemical versus electromagnetic free energy.

This manuscript’s primary focus is on how temporal strategies increases entropy production over time-agnostic strategies. Temporal strategies are one of the hallmarks of biology, such as circadian clocks [60], anticipatory control [34,35], energy and resource storage [32,33], dormancy and persister cells [36], and resource time sharing [61]. More recently, microbial communities have been found to exhibit circadian dynamics as well [31,62,63], but other than passive storage [47,64], marine biogeochemistry models typically do not include such mechanisms. Our results show that including temporal strategies results in significantly greater entropy production than balanced growth, and that explicit strategies, such as the simple diel square-wave function, increase entropy production further over passive strategies, especially near the upper limits of phytoplankton growth. These results are consistent with observations that show how bacteria shift cellular metabolic function to cope with fluctuating environments [65] and how phytoplankton and other members of the community change metabolic expression over diel cycles [30,62,66]. There are system biology models that have been constructed for phytoplankton that show the importance of time-dependent resource allocation [67], but these are not at an ecosystems scale, which is the focus of our study. The ability of biology to detect its environment is also critical to selection of strategies [28], but we have not incorporated that aspect here.

The MEP model using the circadian temporal strategy also makes some predictions regarding specific growth rate and C:N composition of phytoplankton, although we did not explore these areas in detail. The simulations indicate a maximum phytoplankton growth rate around 2 d^−1^ before washout occurs, which is near observed maximum phytoplankton growth rates at 20 °C [68]. These results are encouraging in that our formulation does not include a parameter for maximum specific growth rate like standard kinetic models, but only uses photon interception rate combined with an MEP-determined trait on growth efficiency, $\varepsilon_{P\left\{i\right\}}$, to set the specific growth rate. We have not included temperature dependency in the MEP model, so we cannot compare our results to the full Eppley curve [69], but adding temperature dependency is a next step in model development. The addition of the phytoplankton carbon storage improves growth rate for both the passive and circadian strategies over the balanced growth strategy, which is consistent with observation on improving competitive advantage in fluctuating environments [67,70].

The model also predicts phytoplankton C:N ratio, which varies between 140:1 and 270:1 for the nominal simulation, which is considerably higher than the Redfield ratio of 6.6:1. The C:N ratio for phytoplankton is known to vary as a function of growth rate and nutrient limitations, but the maximum observed values are closer to 50:1 [71,72,73]. In the MEP simulations, the high levels of internal carbon storage in both the passive and circadian strategies is used to capture and dissipate light to enhance entropy production, which differs from how storage is typically viewed, such a survival in fluctuating environments [70]. The internal C storage, $C_{P\left\{i\right\}}$, behaves as a particle in the light attenuation model (Equation (S8)); consequently, one mechanism to dissipate electromagnetic radiation when N is limiting is to increase $C_{P\left\{i\right\}}$ concentration, which is what the passive and circadian strategies implement since there is no model constraint on the C:N ratio of phytoplankton. Effectively, the high phytoplankton C:N ratio is a consequence of the light attenuation model used. As far as we know, there have not been studies that examine how light attenuation changes with phytoplankton internal carbon stores, so our first-order approximation assumed a linear relationship between the light attenuation factor, $k_{wp}$, and the concentration of phytoplankton carbon storage, $\left[C_{P\left\{i\right\}}\right]$. Since a linear formulation results in excessively high phytoplankton C:N ratios, that assumption should be revisited in future versions of the MEP model, which we discuss at the end of this section.

Simulations with the more complex food webs, 2P2B2C and 3P3B3C, did not show much improvement in entropy production when specific growth rates were low (0.2 d^−1^), but they did produce more entropy at higher specific growth rates (1.5 and 2.0 d^−1^) compared to the 1P1B1C food web configuration. At the higher specific growth rates, the more complex models discovered complementarity [74,75], where trait values for one phytoplankton specialized in high light intensity during the summer and another ecotype had trait values that performed better under winter conditions. Complementarity is also exhibited in trait-based models [76] provided the initial population contains ecotypes with diverse parameterizations. In our approach, this is not necessary as the optimization sets the trait values and will select for complementary ecotypes when the strategy increases entropy production.

The optimization approach also provides a potential solution to food web closure. In standard compartment models as well as trait-based models, there is often uncertainly on the number of trophic levels that should be included in the model, and it has been demonstrated that the type of closure, which refers to a top predator that is not formally included as a state variable in the model, dramatically changes simulation results [77,78,79]. In most natural environments, there is usually some limiting resource, such as energy input or chemical constituent. From an optimization perspective, how should the limiting resource, such as N, be allocated to phytoplankton versus bacteria versus consumers or viruses? In our formulation, the structure of the trophic levels described by the matrix $\Omega_{\chi \left\{j\right\},C\left\{i\right\}}$ is determined as part of the optimization. In Section 3.3, the optimizations located three general attractors, the first being a solution that did not allocate much resources to the consumers (bacteria only), while the second optimum attractor did allocate resources to consumers, which resulted in greater entropy production. While often underappreciated, predators can increase the growth rate of their prey by increasing recycling of nutrients that limit prey growth [80,81,82,83]. In essence, the addition of predators increases the rate at which a system cycles, and since the cycle is powered by free energy dissipation, the presence of predators can lead to increase entropy production, as was found by the second optimum attractor in our study. Predation does enhance entropy production.

Casting the model in the currency of entropy production allows comparison between abiotic and biotic processes as well as comparison of different free energy sources driving system organization. The input concentrations in the nominal simulations reflect values found in natural systems, but by casting the model in entropy production, or free energy input, it revealed that solar radiation dominated energy input compared to chemical free energy by more than a 1000-fold. When the model was rerun with near equal inputs of electromagnetic and chemical free energy, three different attractors where identified consisting of bacteria only, bacteria with consumer predation, and phytoplankton only. While the latter solution was found to be the global maximum, the other solutions were locally stable. Furthermore, while we mostly presented only the best solution from the 90 optimizations run, other solutions that were near the maximum entropy production were also found, and some of these solutions exhibited different dynamics of the functional groups or chemical species. In fact, one of the requirements for systems to follow MEP trajectories is that there must be multiple degrees of freedom in the system, and that there are often many solutions that can generate equivalent entropy production [3]. Examining local optima revealed by the MEP model may shed light on ecosystem stability and tipping points, as we would expect an ecosystem to shift over time to higher entropy producing states, especially if new states arise due to environmental change or from sufficiently large perturbations [84,85,86].

We end this section with a few research directions for entropy guided trait-based modeling. All simulations explored in this manuscript examined steady state inputs, except for solar radiation that included diel and seasonal variations. Because of the stability of the inputs, complex food webs involving many ecotypes of each functional group did not provide much improvement in entropy production in low growth scenarios; however, if simulations were run with time varying inputs or step changes, we would expect the higher number of environmental niches would drive optimal solutions to exhibit complementarity. For instance, solutions would likely include ecotypes with oligotrophic or copiotrophic growth kinetics, or high light versus low light ecotypes, if those niches were present during the simulation. Consequently, it might be possible to conduct trait-based optimization in 0D to develop food webs capable of high entropy production under several different environmental conditions. Because the optimizations are computationally expensive, conducting them in 0D environments would greatly increase speed of determining trait values for optimum food webs that are able to exploit complex niches. Once determined, the optimized food web could be run in 3D global circulation models without the computationally costly optimization component.

Another area that needs advancing is the light attenuation model. We only examined blue light, and the light attenuation model (Equation (S8)) is rather simplistic. It is known that phytoplankton can significantly change their light attenuation characteristics of chlorophyll by at least an order of magnitude, and attenuation characteristics vary as a function of wavelength as well [42]. It seems likely that there are energy and resource use tradeoffs in synthesizing different types of light harvesting pigments, but those relationships are not well known. Consequently, exploring resource allocation and light harvesting is needed, since electromagnetic free energy is the dominant input to many ecosystems.

Using information for genome scale metabolic network models [87] could also be useful in defining the reactions used in the distributed metabolic network for the trait-based model, and the reactions governing how consumers remineralize resources as labile versus recalcitrant materials (Equation (S51)) is in need of further research. Perhaps the most interesting question, though, is what are the time scales over which biological strategies operate? What temporal strategies has biology learned over 3.5 billion years of evolution to facilitate entropy production under the guise of Darwinian individual fecundity? Comparing MEP-based simulations to experiments and observations may be one means of answering these questions.

## 5. Conclusions

Under the hypothesis that biological systems organize to dissipate free energy over a characteristic time scale, we have investigated how three temporal strategies affect entropy production in a simple marine food web model consisting of phytoplankton, bacteria, and consumer functional groups. The balanced growth strategy, where phytoplankton grow with fixed stoichiometry, was found to produce the least amount of entropy because growth can only occur when light is present. A significant increase in entropy production occurred with a passive storage strategy that allowed phytoplankton to accumulate reduced carbon during the day to fuel phytoplankton growth in day and night. The best solution, however, was attained by including an explicit circadian clock that dynamically allocated resources to energy harvesting versus biosynthesis reactions to optimally use diel input of solar radiation. We also demonstrated a new type of trait-based modeling that used entropy production maximization to determine trait values as opposed to the standard method of allowing in silico selection to cull the population of poor performers. Our results illustrate that organisms that evolve the ability to anticipate future conditions via explicit temporal strategies can increase entropy production over those that do no. The time scale over which biological systems have evolved to operate, however, remains an open but important question.

## Figures and Tables

**Table 1:** Reactions associated with the three functional groups (phytoplankton (P), bacteria (B), and consumers (C) ), where $R_{j,\chi \left\{i\right\}}$ represents sub-reaction j of biological catalyst ${𝕊}_{\chi \left\{i\right\}}$, and χ{i} is ecotype {i} of P, B, or C, while the rate of reaction $R_{j,\chi \left\{i\right\}}$ is symbolized $r_{j,\chi \left\{i\right\}}$. For consumers, i spans all P{i}, B{i} and C{i}; consequently, among the three functional groups there are a total of $2n_{P}+3n_{B}+\left(n_{P}+n_{B}+n_{C}\right)n_{C}$ reactions. Reactions are shown here to emphasize function only, and the third column shows which functional group catalyzes the reaction. Complete reaction stoichiometries are provided in Section S2.2 of the Supplementary Material and $H_{3}{\mathrm{PO}}_{4}$ and $P_{D}$ (detrital $H_{3}{\mathrm{PO}}_{4}$ ) are only used in thermodynamic calculations but are not state variables in the model. Definitions: $H_{2}CO_{3}$, dissolved inorganic carbon; $\gamma_{H}$, high-energy photons; $C_{P\left\{i\right\}}$, internal carbon storage of phytoplankton; $C_{L}$, labile organic carbon (glucose on unit carbon basis); $C_{D}$, detrital carbon (glucose on unit carbon basis); $N_{D}$, detrital nitrogen (composition as ${\mathrm{NH}}_{3}$ ).

Rxn.	Abbreviated Stoichiometry	Cat.
$R_{1,P\left\{i\right\}}$	$H_{2}{\mathrm{CO}}_{3}+\gamma_{H}\to C_{P\left\{i\right\}}+O_{2}$	${𝕊}_{P\left\{i\right\}}$
$R_{2,P\left\{i\right\}}$	$C_{P\left\{i\right\}}+{\mathrm{NH}}_{3}+H_{3}{\mathrm{PO}}_{4}+O_{2}\to {𝕊}_{P\left\{i\right\}}+H_{2}{\mathrm{CO}}_{3}$	${𝕊}_{P\left\{i\right\}}$
$R_{1,B\left\{i\right\}}$	$C_{L}+{\mathrm{NH}}_{3}+H_{3}{\mathrm{PO}}_{4}+O_{2}\to {𝕊}_{B\left\{i\right\}}+H_{2}{\mathrm{CO}}_{3}$	${𝕊}_{B\left\{i\right\}}$
$R_{2,B\left\{i\right\}}$	$C_{D}\to C_{L}$	${𝕊}_{B\left\{i\right\}}$
$R_{3,B\left\{i\right\}}$	$N_{D}\to {\mathrm{NH}}_{3}$	${𝕊}_{B\left\{i\right\}}$
$R_{\chi \left\{j\right\},C\left\{i\right\}}$	${𝕊}_{\chi \left\{j\right\}}+C_{\chi \left\{j\right\}}+O_{2}\to {𝕊}_{C\left\{i\right\}}+H_{2}{\mathrm{CO}}_{3}+C_{D}+{\mathrm{NH}}_{3}+N_{D}+H_{3}{\mathrm{PO}}_{4}+P_{D}$	${𝕊}_{C\left\{i\right\}}$

**Table 2:** Concentrations of state variables in the feed, as well as environmental conditions for the nominal simulations, where $I_{s}$ is ionic strength and $I_{0}^{M}$ is the maximum surface solar radiation at 0° latitude.

Input	Value	Input	Value
$I_{0}^{M}⟦mmol-\gamma \;m^{-2}\;d^{-1}⟧$	406,000	$\left[C_{L}\right]⟦mmol\;m^{-3}⟧$	10
T⟦K⟧	293	$\left[C_{D}\right]⟦mmol\;m^{-3}⟧$	100
pH	8.1	$\left[N_{D}\right]⟦mmol\;m^{-3}⟧$	7
$I_{s}⟦M⟧$	0.72	$\left[{𝕊}_{P\left\{i\right\}}\right]⟦mmol\;m^{-3}⟧$	0.1
$\left[H_{2}CO_{3}\right]⟦mmol\;m^{-3}⟧$	2000	$\left[C_{P\left\{i\right\}}\right]⟦mmol\;m^{-3}⟧$	0.1
$\left[O_{2}\right]⟦mmol\;m^{-3}⟧$	225	$\left[{𝕊}_{B\left\{i\right\}}\right]⟦mmol\;m^{-3}⟧$	0.1
$\left[NH_{3}\right]⟦mmol\;m^{-3}⟧$	5	$\left[{𝕊}_{C\left\{i\right\}}\right]⟦mmol\;m^{-3}⟧$	0.1

**Table 3:** Optimal trait values obtained from maximizing total entropy production, $\sigma^{T}$, in a 1P1B1C food web model over a two-year period for the three different temporal strategies under nominal conditions (Table 2) at a dilution rate of 0.2 d^−1^.

Variable	Balanced Growth	Passive Storage	Circadian Allocation
$\varepsilon_{P\left\{1\right\}}$	0.2536	0.3452	0.3788
$t_{On\left\{1\right\}}\;\left(d\right)$	0.0000 *	0.0000 *	0.2389
$t_{Off\left\{1\right\}}\;\left(d\right)$	1.0000 *	1.0000 *	0.7799
$\Omega_{amp\left\{1\right\}}$	0.6746	0.8036	1.0000
$\varepsilon_{B\left\{1\right\}}$	0.1686	0.1618	0.1628
$\Omega_{1,B\left\{1\right\}}$	0.3351	0.3852	0.4349
$\Omega_{2,B\left\{1\right\}}$	0.1997	0.1609	0.2869
$\Omega_{3,B\left\{1\right\}}$	0.4651	0.4539	0.2782
$\varepsilon_{C\left\{1\right\}}$	0.0001	0.9971	0.0001
$\Omega_{P\left\{1\right\},C\left\{1\right\}}$	0.7288	0.0000	0.6547
$\Omega_{B\left\{1\right\},C\left\{1\right\}}$	0.0729	0.3888	0.5809
$\Omega_{C\left\{1\right\},C\left\{1\right\}}$	0.4396	0.5868	0.0963
$\sigma^{R}\;\left(MJ\;K^{-1}\right)$	0.2826	0.9901	0.9787
$\sigma^{W}\;\left(MJ\;K^{-1}\right)$	0.2751	1.687	1.616
$\sigma^{P}\;\left(MJ\;K^{-1}\right)$	3.426	17.79	18.60
$\sigma^{T}\;\left(MJ\;K^{-1}\right)$	3.984	18.95	19.74

* These values were held constant and not part of the optimization.

**Table 4:** Total entropy production, $\sigma^{T}$, from three different food web configurations over a two-year period with the three different temporal strategies under nominal conditions (Table 2) at a dilution rate of 0.2 d^−1^.

Strategy	1P1B1C	2P2B2C	3P3B3C
**Balanced**	3.9837	4.034	4.0811
**Passive**	18.9511	18.9976	19.0152
**Circadian**	19.7359	19.7844	19.7987

**Table 5:** Total entropy production, $\sigma^{T}$, from three different food web configurations over a two-year period, each run using the three different temporal strategies under nominal inputs concentrations (Table 2) but at a dilution rate of 1.5 d^−1^.

Strategy	1P1B1C	2P2B2C	3P3B3C
**Balanced**	0.3226	0.3455	0.3684
**Passive**	1.3374	1.4995	1.6432
**Circadian**	3.0891	3.4000	3.6242

## Supplementary Materials

The following are available online at https://www.mdpi.com/1099-4300/22/11/1249/s1, Governing Equations (S1)–(S82) with Figure S1 and an example parameter input file.
