# Supplementary material for: Phytoplankton Temporal Strategies Increase Entropy Production in a Marine Food Web Model
Source: Entropy (Basel). 2020 Nov 3;22(11):1249. doi: 10.3390/e22111249 (PMC7712749; doi:10.3390/e22111249)
Supplement: Supplementary file 1 [file entropy-22-01249-s001.pdf]

Supplementary Material

Phytoplankton temporal strategies increase entropy production in marine a food web model

Joseph Vallino [1]*and Ioannis Tsakalakis [2]

[1] Marine Biological Laboratory; jvallino@mbl.edu
[2] Marine Biological Laboratory and Massachusetts Institute of Technologhy; itsakalakis@mbl.edu
* Correspondence: jvallino@mbl.edu

Received: date; Accepted: date; Published: date

S1. Overview

This *Supplementary Material* describes the details of the model used to demonstrate how temporal strategies can increase entropy production over a given time interval. Constituent transport is governed by a simple well-mixed chemostat-like pond of constant volume that receives a constant flow of water with defined input concentrations and is illuminated at the surface with monochromatic light (blue, 440 nm) that varies on both diel and seasonal cycles. The food web consists of three functional groups, phytoplankton, $\mathbb{S}_P$, bacteria, $\mathbb{S}_B$, and consumers, $\mathbb{S}_C$, that produce or consume dissolved inorganic carbon, H_2CO_3, oxygen, O_2, ammonium, NH_3, labile organic carbon, C_L, and detrital organic carbon and nitrogen, C_D and N_D, respectively (Figure 1). Biological structures for all three functional groups are given the same unit-carbon elemental composition, $CH_{\alpha_\mathbb{S}}O_{\beta_\mathbb{S}}N_{\gamma_\mathbb{S}}P_{\delta_\mathbb{S}}$, but phytoplankton also contain an internal pool of carbon, C_P, with elemental composition CH_2O. All concentrations are in $[\![mmol\, m^{-3}]\!]$, where double brackets are used to indicate units of variables. The model uses a trait-based approach [1] where each functional group, $\mathbb{S}_{\chi\{i\}}$, is represented by n_χ ecotypes, or realizations, that are assigned different parameter values that govern reaction stoichiometries, growth kinetics and protein allocation to metabolic pathways, where χ is either P, B or C. Unlike canonical trait-based models, parameters governing traits (aka control variables [2]) for each ecotype are not randomly assigned but determined by solving a non-linear optimization problem that maximizes integrated entropy production associated with irreversible processes over a fixed simulation period of two years.

S2. Transport, Reaction and Entropy Production Model

S2.1 Mass Balance model

The maximum entropy production (MEP)-optimize trait-based model uses a simple 0D, well-mixed system for transport, where nutrients and low concentrations of organisms flow into a reservoir of volume $V\,[\![m^3]\!]$ at flow rate $F\,[\![m^3\,d^{-1}]\!]$ to produce a dilution rate of $D[\![d^{-1}]\!]=\frac{F}{V}$. The pond-like cylindrical reservoir is in contact with the atmosphere at one end, has a cross-sectional area $A\,[\![m^2]\!]$ and depth $\zeta_d\,[\![m]\!]$ and is illuminated at the surface with photosynthetically active radiation (PAR) of intensity $I_0(t)\,[\![mmol\, photons\, m^{-2}\, d^{-1}]\!]$ that varies both diurnally and seasonally [3]. A simple mass balance around the state variables leads to an initial value problem, which, in vector form, is as follows,

$$\frac{d\mathbf{c}(t)}{dt}=D(\mathbf{c}^I-\mathbf{c}(t))+\frac{A}{V}\mathbf{v}\circ\left(\mathbf{p}\circ\mathbf{h}(T)-\mathbf{c}(t)\right)+\mathbf{S}(\mathbf{u})\mathbf{r}(t;\mathbf{u});\quad \left.\frac{d\mathbf{c}(t)}{dt}\right|_{t=t_0}=\mathbf{c}^I, \tag{S1}$$

where $\mathbf{c}(t) \in \mathbb{R}^{ns}$ is a state vector of n_S concentration variables $[\![mmol\, m^{-3}]\!]$ given by,

$$\mathbf{c}^T(t) = \begin{bmatrix} c_{H_2CO_3}\; c_{O_2}\; c_{NH_3}\; c_{C_L}\; c_{C_D}\; c_{N_D}\; c_{\mathbb{S}_{P\{1\}}}, \dots, c_{\mathbb{S}_{P\{n_P\}}}\; c_{C_{P\{1\}}}, \dots, c_{C_{P\{n_P\}}} \\ c_{\mathbb{S}_{B\{1\}}}, \dots, c_{\mathbb{S}_{B\{n_B\}}}\; c_{\mathbb{S}_{C\{1\}}}, \dots, c_{\mathbb{S}_{C\{n_C\}}} \end{bmatrix}, \tag{S2}$$

that consists of 6 chemical constituents, n_P phytoplankton ecotypes with associated n_P internal C_P storage pool, n_B bacteria ecotypes and n_C consumer ecotypes so that $n_S = 6 + 2n_P + n_B + n_C$; $\mathbf{c}^I$ are the input concentrations that also serve as the initial conditions at t_0; a stagnant-film model governs mass exchange across the air-water interface for state variables with gas phases (CO_2 and O_2), where $\mathbf{v}\,[\![m\, d^{-1}]\!]$ is the piston velocity, $\mathbf{p}\,[\![Pa]\!]$ is atmospheric gas partial pressure and $\mathbf{h}(T)$ is the Henry's law coefficient $[\![mmol\, m^{-3}\, Pa^{-1}]\!]$ at temperature $T\,[\![K]\!]$, and $\circ$ is the element-wise multiplication (Hadamard) operator; $\mathbf{r}(t;\mathbf{u}) \in \mathbb{R}^{n_r}$ is a vector of n_r reaction rates $[\![mmol\, m^{-3}\, d^{-1}]\!]$ associated with biological structures (see below) and $\mathbf{S}(\mathbf{u}) \in \mathbb{R}^{n_{\mathbb{S}} \times n_r}$ is a reaction stoichiometry matrix. Reaction rates and the stoichiometric matrix depend on a time-invariant control vector, $\mathbf{u}$, that consists of a vector of reaction efficiencies, $\boldsymbol{\varepsilon}$, and a vector of resource allocation controls, $\boldsymbol{\Omega}$, ($\mathbf{u}^T = [\boldsymbol{\varepsilon}^T\; \boldsymbol{\Omega}^T]$) described in Section S2.2. In this formulation, the control variables, $\boldsymbol{\Omega}$ and $\boldsymbol{\varepsilon}$, are held constant for each ecotype, so serve as the trait variables.

S2.2 Metabolic Reaction Rates

The metabolic reactions associated with phytoplankton, bacteria and consumers (grazers) follows that developed previously [2], except in this implementation each functional group can have a specified number of ecotypes that have different values for the control variables (i.e., traits). We use braces, $\{i\}$, to designate each realization of an ecotype defined by the trait values and χ represents one of the three functional groups (P, B or C). The governing equations for each of the three functional groups are given below, with the following overall organization. The metabolic reactions a functional group is capable of catalyzing includes a thermodynamic efficiency trait, $\varepsilon_{\chi\{i\}}$, that specifies weighting between an anabolic (i.e., biosynthesis) reaction and a catabolic (energy producing) reaction. The anabolic and catabolic reactions are combined into a single reaction and balanced with the parameter $n_{j,\chi\{i\}}$ that ensures as $\varepsilon_{\chi\{i\}}$ approaches 1, the Gibbs free energy of reaction goes to 0, so the reaction is at equilibrium. The anabolic and catabolic reactions given below can be recovered by setting $n_{j,\chi\{i\}}$ to 0 and setting $\varepsilon_{\chi\{i\}}$ to 1 or 0, respectively. Reaction entropy is maximized as $\varepsilon_{\chi\{i\}}$ approaches 0, as this represents complete conversion of free energy to heat. Stoichiometric coefficients, such as $a_{j,P}^A$ and $b_{j,B}^C$, are used to balance O and H, respectively, where the superscript is for either the anabolic (A) or catabolic (C) reaction, and the subscripts correspond to the reaction number, j, for the associated functional group (P, B or C). Defined by whole reaction stoichiometry, the Gibbs free energy of reaction, $\Delta_r G$, accounts for the reaction quotient, and the standard Gibbs free energy of reaction, $\Delta_r G^o$, is obtained from Alberty's [4] that accounts for ionization of chemical species based on pH and temperature, and ionic strength is used to approximate activity from concentration. Reaction kinetics are based on an adaptive Monod equation [2] that consists of a kinetic driver, F_K, that is parameterized by $\varepsilon_{\chi\{i\}}$ and also includes a thermodynamic driver, F_T, that depends on the number of electrons, $n_{j,\chi\{i\}}^e$, transferred in the catabolic reaction as described by LaRowe et al. [5]. Bracket notation, [ ], is used to represent concentration of state variables (i.e., $[NH_3]$ and c_{NH_3} are equivalent). The fraction of biological structure allocated to each metabolic reaction that a functional group can catalyzed is determined by $\Omega_{j,\chi\{i\}}$, where $\sum_j \Omega_{j,\chi\{i\}} = 1$ and $0 \le \Omega_{j,\chi\{i\}} \le 1\; \forall\, j$ because the total catalytic capacity is constrained by the concertation of biological structure, $[\mathbb{S}_{\chi\{i\}}]$, that changes over time. Entropy production is calculated for dissipation of chemical free energy by metabolic reactions, $\dot{\sigma}_{j,\chi\{i\}}^R$, as well as dissipation of electromagnetic free energy by particulate material, $\dot{\sigma}_{j,\chi\{i\}}^P$, and water, $\dot{\sigma}^W$, although the latter does not depend on any of the state variables, so is not listed below (See Section S2.4 below). We use $R_{j,\chi\{i\}}$

to refer to the stoichiometry of reaction j catalyzed by biological structure $\mathbb{S}_{\chi\{i\}}$, and $r_{j,\chi\{i\}}$ for the reaction rate.

S2.2.1 Phytoplankton Reactions

Phytoplankton are represented with two metabolic reactions consisting of 1) $R_{1,P\{i\}}$, CO_2 fixation into unit-C sugar (i.e. CH_2O, or $C_{P\{i\}}$) driven by high frequency photon, γ_H, capture and 2) $R_{2,P\{i\}}$, conversion of $C_{P\{i\}}$ into biomass using available ammonium and phosphate driven by the catabolic aerobic oxidation of $C_{P\{i\}}$. (Note, phosphate is not a state variable and is held at a fixed concentration of 1 μM during simulations.) Surficial light intensity, $I_0(t)$, varies on both diel and seasonal cycles [3], and depth-average light intensity, $\langle I(t)\rangle_{\zeta_d}$, for the well-mixed system is calculated from $I_0(t)$ and light at depth ζ_d, where light attenuation occurs by water, particle and chlorophyll a absorption as parameterized by k_w, k_p and k_{Chl} respectively. We only consider blue light at 440 nm and the light attenuation coefficients, k_w, k_p and k_{Chl} were derived from Wozniak [6] and set to 0.011 m^{-1}, 0.000625 m^2 (mmol-C)$^{-1}$ and 0.0025 m^2 (mmol-C)$^{-1}$ for 440 nm light, respectively, after conversion to mM C. The Gibbs free energy of photons, $\Delta_r G_\gamma$, at 440 nm is -253 J (mmol- γ)$^{-1}$, which accounts for the conversion of photons to work [2,7]. Entropy production for phytoplankton is divided into reaction associated, $\dot{\sigma}^R_{1,P\{i\}}$, and particle absorption, $\dot{\sigma}^P_{1,P\{i\}}$, components that are controlled by $\Omega_{1,P\{i\}}$ and $\Omega_{2,P\{i\}}$. Light absorbed by water and non-photosynthetic biomass, $\Omega_{2,P\{i\}}[\mathbb{S}_{P\{i\}}]$, is simply dissipated as heat and contributes to entropy production. Only the fraction of electromagnetic free energy that is converted to chemical potential (i.e., $C_{P\{i\}}$ and $\mathbb{S}_{P\{i\}}$ synthesis) does not contribute to entropy production (see Eqn. (S13) for $\dot{\sigma}^T_{1,P\{i\}}$ below). The fraction of phytoplankton biomass allocated to photosynthetic processes described by $R_{1,P\{i\}}$ is given by $\Omega_{1,P\{i\}}[\mathbb{S}_{P\{i\}}]$; however, since the photosynthetic machinery can be kinetically limited by resource availability $(i.e., [CO_2] + [HCO_3^-])$ or thermodynamics, F_T, only the fraction of the total photon capture rate that contributes to $r_{1,P\{i\}}$, given by $\frac{\Delta I_{P\{i\}}}{n_{1,P\{i\}}}$, contributes to $\dot{\sigma}^R_{1,P\{i\}}$, while the remainder contributes to $\dot{\sigma}^P_{1,P\{i\}}$. That is, if the photosynthetic machinery is constrained, the excess light captured is dissipated as heat, so contributes to $\dot{\sigma}^P_{1,P\{i\}}$. Light intercepted by biological structure allocated to biosynthesis, given by $\Omega_{2,P\{i\}}[\mathbb{S}_{P\{i\}}]$, always contributes to particle-associated entropy production, while dissipation of chemical free energy associated with catabolic reactions contributes to reaction-associated entropy production, $\dot{\sigma}^R_{2,P\{i\}}$. For the carbon dioxide fixation reaction ($R_{1,P\{i\}}$, Eq. (S3)), $n_{1,P\{i\}}$ is the moles of high frequency photons, γ_H, needed to fix one mole of CO_2, reversibly, under the current conditions, so that the quantum yield, $\frac{n_{1,P\{i\}}}{\varepsilon_{P\{i\}}}$, depends on $\varepsilon_{P\{i\}}$. The concentration of fixed carbon, $[C_{P\{i\}}]$, is based on total system volume, but for kinetics it is treated as an intracellular component, so is multiplied by a system-to-cell volume factor, φ_f, to reflect its higher intracellular concentration (φ_f was set to 1000 for all simulations). Below are the equations describing phytoplankton growth and associated entropy production.

<u>Carbon dioxide fixation driven by solar radiation</u>: $R_{1,P\{i\}}$

Overall stoichiometry of reaction $R_{1,P\{i\}}$ containing catabolic and anabolic sub-reactions,

$$R_{1,P\{i\}}: \varepsilon_{P\{i\}}\mathrm{H_2CO_3} + n_{1,P\{i\}}\gamma_H \rightarrow \varepsilon_{P\{i\}}\left(\mathrm{C}_{P\{i\}} + \mathrm{O_2}(aq)\right). \tag{S3}$$

The free energy needed to fix $\mathrm{H_2CO_3}$ into $\mathrm{C}_{P\{i\}}$ (unit carbon glucose) and $\mathrm{O_2}$ (anabolic sub-reaction) is given by,

$$\Delta_r G^o_{C_{P\{i\}}} = \Delta_f G^o_{CH_2O} + \Delta_f G^o_{O_2(aq)} - \Delta_f G^o_{H_2CO_3}, \tag{S4}$$

$$\Delta_r G_{C_{P\{i\}}} = \Delta_r G^o_{C_{P\{i\}}} + RTln\left(\frac{[\mathrm{C}_{P\{i\}}][\mathrm{O_2}(aq)]}{[\mathrm{H_2CO_3}]}\right). \tag{S5}$$

The moles of photons with free energy $\Delta_r G_\gamma$ needed to produce one mole of $\mathrm{C}_{P\{i\}}$ is given by,

$$n_{1,P\{i\}} = -\frac{\Delta_r G_{C_{P\{i\}}}}{\Delta_r G_\gamma}, \tag{S6}$$

where $n_{1,P\{i\}}/\ \varepsilon_{P\{i\}}$ is the quantum yield of photosynthesis.

The overall Gibbs free energy of reaction for $R_{1,P\{i\}}$ is then,

$$\Delta_r G_{1,P\{i\}} = -\left(1 - \varepsilon_{P\{i\}}\right)\Delta_r G_{C_{P\{i\}}}. \tag{S7}$$

Light attenuation by water, particles and photosynthetic machinery (Chl a) is given by,

$$\begin{aligned} k_{wp} = k_w + k_p \left(\sum\nolimits_i \Omega_{2,P\{i\}}\left[\mathbb{S}_{P\{i\}}\right] + \sum\nolimits_i \left[C_{P\{i\}}\right] + \sum\nolimits_i \left[\mathbb{S}_{B\{i\}}\right] + \sum\nolimits_i \left[\mathbb{S}_{C\{i\}}\right]\right) \\ + k_{Chl} \sum\nolimits_i \Omega_{1,P\{i\}}\left[\mathbb{S}_{P\{i\}}\right]. \end{aligned} \tag{S8}$$

Average light intensity in a well-mixed reservoir of depth ζ_d is given by,

$$\langle I(t)\rangle_{\zeta_d} = \frac{I_0(t)\left(1 - e^{-k_{wp}\zeta_d}\right)}{k_{wp}\zeta_d}, \tag{S9}$$

and the volumetric rate of photon capture by Chl a over depth ζ_d is,

$$\Delta I_{P\{i\}} = k_{Chl}\Omega_{1,P\{i\}}\left[\mathbb{S}_{P\{i\}}\right]\langle I(t)\rangle_{\zeta_d}. \tag{S10}$$

Electrons transfer in the catabolic sub-reaction of $R_{1,P\{i\}}$ is

$$n^e_{1,P\{i\}} = n_{1,P\{i\}}. \tag{S11}$$

Reaction rate of $R_{1,P\{i\}}$ is given by,

$$r_{1,P\{i\}} = \frac{\Delta I_{P\{i\}}}{n_{1,P\{i\}}}\left(\frac{[\mathrm{CO_2}] + [\mathrm{HCO_3^-}]}{[\mathrm{CO_2}] + [\mathrm{HCO_3^-}] + \kappa^* \varepsilon^4_{P\{i\}}}\right) F_T\left(\Delta_r G_{1,P\{i\}}, n^e_{1,P\{i\}}\right), \tag{S12}$$

where the term enclosed by ( ) is the kinetic drive, F_K. Total entropy production from reaction $R_{1,P\{i\}}$ and light absorption by photosynthetic apparatus of $\mathbb{S}_{P\{i\}}$ is given by,

$$\dot{\sigma}^T_{1,P\{i\}} = \frac{A\zeta_d}{T}\Delta_r G_{C_{P\{i\}}}\left(\frac{\Delta I_{P\{i\}}}{n_{1,P\{i\}}} - \varepsilon_{P\{i\}} r_{1,P\{i\}}\right), \tag{S13}$$

which contributes to entropy production from reactions and particles as follows,

$$\dot{\sigma}^R_{1,P\{i\}} = r_{1,P\{i\}}\frac{n_{1,P\{i\}}}{\Delta I_{P\{i\}}}\dot{\sigma}^T_{1,P\{i\}}, \tag{S14}$$

$$\dot{\sigma}^P_{1,P\{i\}} = \dot{\sigma}^T_{1,P\{i\}} - \dot{\sigma}^R_{1,P\{i\}}. \tag{S15}$$

Note, if $r_{1,P\{i\}}$ is unconstrained by F_K or F_T, then $\dot{\sigma}^R_{1,P\{i\}} = \dot{\sigma}^T_{1,P\{i\}}$ and $\dot{\sigma}^P_{1,P\{i\}} = 0$.

<u>Conversion of fixed carbon into phytoplankton biomass</u>: $R_{2,P\{i\}}$

Full stoichiometry of reaction $R_{2,P\{i\}}$ is given by,

$$\begin{aligned} \left(1 + \varepsilon_{P\{i\}} n_{2,P\{i\}}\right)\mathrm{C}_{P\{i\}} + \varepsilon_{P\{i\}}(\gamma_{\mathbb{S}}\mathrm{NH_3} + \delta_{\mathbb{S}}\mathrm{H_3PO_4}) + \left(1 + \varepsilon_{P\{i\}}\left(a^A_{2,P} + n_{2,P\{i\}} - 1\right)\right)\mathrm{O_2}(aq) \\ \rightarrow \varepsilon_{P\{i\}}\mathbb{S}_{P\{i\}} + \varepsilon_{P\{i\}} b^A_{2,P}\mathrm{H_2O} + \left(1 + \varepsilon_{P\{i\}}\left(n_{2,P\{i\}} - 1\right)\right)\mathrm{H_2CO_3}, \end{aligned} \tag{S16}$$

where the stoichiometric coefficients to balance H and O are,

$$a^A_{2,P} = \tfrac{1}{4}(-\alpha_{\mathbb{S}} + 2\beta_{\mathbb{S}} + 3\gamma_{\mathbb{S}} - 5\delta_{\mathbb{S}}), \tag{S17}$$

$$b^A_{2,P} = \tfrac{1}{2}(2 - \alpha_{\mathbb{S}} + 3\gamma_{\mathbb{S}} + 3\delta_{\mathbb{S}}). \tag{S18}$$

Free energy of reaction for the anabolic component of $R_{2,P\{i\}}$, $R^A_{2,P\{i\}} \stackrel{\mathrm{def}}{=} \mathrm{C}_{P\{i\}} + \gamma_{\mathbb{S}}\mathrm{NH_3} + \delta_{\mathbb{S}}\mathrm{H_3PO_4} + a^A_{2,P}\mathrm{O_2}(aq) \rightarrow \mathbb{S}_{P\{i\}} + b^A_{2,P}\mathrm{H_2O}$, is given by,

$$\Delta_r^A G_{2,P\{i\}}^o = \left(\Delta_f G_{\mathbb{S}}^o + b_{2,P}^A \Delta_f G_{H_2O}^o\right) - \left(\Delta_f G_{C_{P\{i\}}}^o + \gamma_{\mathbb{S}} \Delta_f G_{NH_3}^o + \delta_{\mathbb{S}} \Delta_f G_{H_3PO_4}^o + a_{2,P}^A \Delta_f G_{O_2(aq)}^o\right), \quad \text{(S19)}$$

$$\Delta_r^A G_{2,P\{i\}} = \Delta_r^A G_{2,P\{i\}}^o + RTln\left(\frac{[\mathbb{S}_{P\{i\}}]}{[\mathrm{C}_{Phy}][\mathrm{NH}_3]^{\gamma_{\mathbb{S}}}[\mathrm{H}_3\mathrm{PO}_4]^{\delta_{\mathbb{S}}}[\mathrm{O}_2(aq)]^{a_{2,P}^A}}\right). \quad \text{(S20)}$$

Free energy of reaction for catabolic component of $R_{2,P\{i\}}$, $R_{2,P\{i\}}^C \stackrel{\text{def}}{=} \mathrm{C}_{P\{i\}} + \mathrm{O}_2(aq) \rightarrow \mathrm{H}_2\mathrm{CO}_3$, is given by,

$$\Delta_r^C G_{2,P\{i\}}^o = \Delta_f G_{H_2CO_3}^o - \left(\Delta_f G_{C_{P\{i\}}}^o + \Delta_f G_{O_2(aq)}^o\right), \quad \text{(S21)}$$

$$\Delta_r^C G_{2,P\{i\}} = \Delta_r^C G_{2,P\{i\}}^o + RTln\left(\frac{[\mathrm{H}_2\mathrm{CO}_3]}{[\mathrm{C}_{P\{i\}}][\mathrm{O}_2(aq)]}\right). \quad \text{(S22)}$$

In order to insure that $R_{2,P\{i\}}$ is at equilibrium when $\varepsilon_{P\{i\}} = 1$, the catabolic reaction, $R_{2,P\{i\}}^C$, is added to the anabolic reaction, $R_{2,P\{i\}}^A$, so that the Gibbs free energy of reaction, $\Delta_r G_{2,P\{i\}}$, for $R_{2,P\{i\}}$ equals zero. The coefficient $n_{2,P\{i\}}$ defines moles of $\mathrm{C}_{P\{i\}}$ that need to be oxidized for this to occur when $\varepsilon_{P\{i\}} = 1$, which is given by:

$$n_{2,P\{i\}} = -\frac{\Delta_r^A G_{2,P\{i\}}}{\Delta_r^C G_{2,P\{i\}}}. \quad \text{(S23)}$$

Consequently, the Gibbs free energy of reaction for $R_{2,P\{i\}}$ is,

$$\Delta_r G_{2,P\{i\}} = \left(1 - \varepsilon_{P\{i\}}\right)\Delta_r^C G_{2,P\{i\}}, \quad \text{(S24)}$$

and the electrons transferred in reaction $R_{2,P\{i\}}^C$ is given by,

$$n_{2,P\{i\}}^e = 4. \quad \text{(S25)}$$

Reaction rate of $R_{2,P\{i\}}$:

$$\begin{aligned} r_{2,P\{i\}} &= \nu^* \varepsilon_{P\{i\}}^2 \Omega_{2,P\{i\}}[\mathbb{S}_{P\{i\}}]\left(\frac{[C_{P\{i\}}]\varphi_f}{[C_{P\{i\}}]\varphi_f + \kappa^* \varepsilon_{P\{i\}}^4}\right)\left(\frac{[\mathrm{NH}_3]/\gamma_{\mathbb{S}}}{[\mathrm{NH}_3]/\gamma_{\mathbb{S}} + \kappa^* \varepsilon_{P\{i\}}^4}\right) \\ &\times \left(\frac{[\mathrm{O}_2]}{[\mathrm{O}_2] + \kappa^* \varepsilon_{P\{i\}}^4}\right) F_T\left(\Delta_r G_{2,P\{i\}}, n_{2,P\{i\}}^e\right), \end{aligned} \quad \text{(S26)}$$

where again the kinetic drive is comprised of the terms in ( ). Entropy production from reaction $R_{2,P\{i\}}$ and light absorption by the biosynthetic fraction of $\mathbb{S}_{P\{i\}}$ biomass is given by,

$$\dot{\sigma}_{2,P\{i\}}^R = -\frac{A\zeta_d}{T} r_{2,P\{i\}} \Delta_r G_{2,P\{i\}}, \quad \text{(S27)}$$

$$\dot{\sigma}_{2,P\{i\}}^P = -\frac{A\zeta_d}{T} \langle I(t)\rangle_d \Delta_r G_\gamma k_p \Omega_{2,P\{i\}}[\mathbb{S}_{P\{i\}}]. \quad \text{(S28)}$$

S2.2.2 Bacteria growth on labile carbon, C_L

Bacteria catalyze three reactions that include growth on labile carbon, C_L, and decomposition of detrital carbon, C_D, and nitrogen, N_D, into labile pools, where $\Omega_{1,B\{i\}}$, $\Omega_{2,B\{i\}}$ and $\Omega_{3,B\{i\}}$ determine the allocation of catalytic machinery to each reaction, respectively. Decomposition of detritus, which is recalcitrant, uses a different biomass-specific rate constant, ν_D^*, than that used for growth on labile carbon. Note, the formulation for $R_{1,B\{i\}}$ differs slightly from that for phytoplankton, $R_{2,B\{i\}}$, in that the anabolic reaction is not exactly balanced by a small amount of the catabolic reaction when $\varepsilon_{B\{i\}} = 1$, so that $\Delta_r G_{1,B\{i\}}$ is slightly less than 0 when $\varepsilon_{B\{i\}} = 1$. Instead, a sufficient amount of labile carbon, C_L, given by $a_{1,B}^A$, is converted to CO_2 to balance O in $R_{1,B\{i\}}$. This slightly different formulation was used to be consistent with previous work [2], but we have found the two different approaches produce the same numerical results.

<u>Bacterial growth</u>: $R_{1,B\{i\}}$

Stoichiometry of reaction $R_{1,B\{i\}}$ and coefficients to balance H and O are given by,

$$\begin{aligned} &\mathrm{C}_L + \varepsilon_{B\{i\}}(\gamma_{\mathbb{S}}\mathrm{NH}_3 + \delta_{\mathbb{S}}\mathrm{H}_3\mathrm{PO}_4) + (1-\varepsilon_{B\{i\}})\mathrm{O}_2(aq) \\ &\quad \rightarrow \varepsilon_{B\{i\}}a_{1,B}^{A}\mathbb{S}_{B\{i\}} + \left(2-\varepsilon_{B\{i\}}\left(a_{1,B}^{A}+1\right)\right)\mathrm{H}_2\mathrm{CO}_3 + \varepsilon_{B\{i\}}b_{1,B}^{A}\mathrm{H}_2\mathrm{O}, \end{aligned} \tag{S29}$$

$$a_{1,B}^{A} = \frac{4+3\gamma_{\mathbb{S}}-5\delta_{\mathbb{S}}}{4+\alpha_{\mathbb{S}}-2\beta_{\mathbb{S}}}, \tag{S30}$$

$$b_{1,B}^{A} = \frac{4-2\alpha_{\mathbb{S}}+9\gamma_{\mathbb{S}}-3\beta_{\mathbb{S}}\gamma_{\mathbb{S}}+\delta_{\mathbb{S}}+4\alpha_{\mathbb{S}}\delta_{\mathbb{S}}-3\beta_{\mathbb{S}}\delta_{\mathbb{S}}}{4+\alpha_{\mathbb{S}}-2\beta_{\mathbb{S}}}. \tag{S31}$$

Gibbs free energy of reaction for anabolic and catabolic components of $R_{1,B\{i\}}$ are,

$$\begin{aligned} &\Delta_r^A G_{1,B\{i\}}^o \\ &\quad = \left(a_{1,B}^{A}\Delta_f G_{\mathbb{S}}^o + \left(1-a_{1,B}^{A}\right)\Delta_f G_{H_2CO_3}^o + b_{1,B}^{A}\Delta_f G_{H_2O}^o\right) \\ &\quad - \left(\Delta_f G_{C_L}^o + \gamma_{\mathbb{S}}\Delta_f G_{NH_3}^o + \delta_{\mathbb{S}}\Delta_f G_{H_3PO_4}^o\right), \end{aligned} \tag{S32}$$

$$\Delta_r^A G_{1,B\{i\}} = \Delta_r^A G_{1,B\{i\}}^o + RTln\left(\frac{\left[\mathbb{S}_{B\{i\}}\right]^{a_{1,B}^{A}}[\mathrm{H}_2\mathrm{CO}_3]^{(1-a_{1,B}^{A})}}{[\mathrm{C}_L][\mathrm{NH}_3]^{\gamma_{\mathbb{S}}}[\mathrm{H}_3\mathrm{PO}_4]^{\delta_{\mathbb{S}}}}\right), \tag{S33}$$

$$\Delta_r^C G_{1,B\{i\}}^o = \Delta_f G_{H_2CO_3}^o - \left(\Delta_f G_{C_L}^o + \Delta_f G_{O_2(aq)}^o\right), \tag{S34}$$

$$\Delta_r^C G_{1,B\{i\}} = \Delta_r^C G_{1,B\{i\}}^o + RTln\left(\frac{[\mathrm{H}_2\mathrm{CO}_3]}{[\mathrm{C}_L][\mathrm{O}_2(aq)]}\right). \tag{S35}$$

Overall Gibbs free energy of reaction for $R_{1,B\{i\}}$ is,

$$\Delta_r G_{1,B\{i\}} = \varepsilon_{B\{i\}}\Delta_r^A G_{1,B\{i\}} + \left(1-\varepsilon_{B\{i\}}\right)\Delta_r^C G_{1,B\{i\}}. \tag{S36}$$

Note, in this formulation $\Delta_r G_{1,B\{i\}}$ equals $\Delta_r^A G_{1,B\{i\}}$ when $\varepsilon_{B\{i\}} = 1$, which is not exactly 0 as was formulated for the phytoplankton biosynthetic reaction, but it is typically close to 0.

Electrons transferred in catabolic portion of reaction $R_{1,B\{i\}}$ are,

$$n_{1,B\{i\}}^{e} = 4, \tag{S37}$$

And the reaction rate, $r_{1,B\{i\}}$, of $R_{1,B\{i\}}$ is,

$$\begin{aligned} r_{1,B\{i\}} &= \nu^{*}\varepsilon_{B\{i\}}^{2}\Omega_{1,B\{i\}}\left[\mathbb{S}_{B\{i\}}\right]\left(\frac{[\mathrm{C_L}]}{[\mathrm{C}_L]+\kappa^{*}\varepsilon_{B\{i\}}^{4}}\right)\left(\frac{[\mathrm{NH}_3]/\gamma_{\mathbb{S}}}{[\mathrm{NH}_3]/\gamma_{\mathbb{S}}+\kappa^{*}\varepsilon_{B\{i\}}^{4}}\right) \\ &\times\left(\frac{[\mathrm{O}_2]}{[\mathrm{O}_2]+\kappa^{*}\varepsilon_{B\{i\}}^{4}}\right)F_T\left(\Delta_r G_{1,B\{i\}}, n_{1,B\{i\}}^{e}\right). \end{aligned} \tag{S38}$$

Entropy production from reaction $R_{1,B\{i\}}$ and light absorption by the fraction of $\mathbb{S}_{B\{i\}}$ biomass allocated to reaction $R_{1,P\{i\}}$ is given by,

$$\dot{\sigma}_{1,B\{i\}}^{R} = -\frac{A\zeta_d}{T}r_{1,B\{i\}}\Delta_r G_{1,B\{i\}} \text{ and} \tag{S39}$$

$$\dot{\sigma}_{1,B\{i\}}^{P} = -\frac{A\zeta_d}{T}\langle I(t)\rangle_d \Delta_r G_\gamma k_p \Omega_{1,B\{i\}}\left[\mathbb{S}_{B\{i\}}\right]. \tag{S40}$$

<u>Bacterial decomposition of recalcitrant carbon</u>: $R_{2,B\{i\}}$

Stoichiometry of reaction $R_{2,B\{i\}}$ is,

$$\mathrm{C}_D \rightarrow \mathrm{C}_L, \tag{S41}$$

where the Gibbs free energy of reaction for $R_{2,B\{i\}}$ due to concentration differences is,

$$\Delta_r G_{2,B\{i\}} = RTln\left(\frac{[\mathrm{C_L}]}{[\mathrm{C}_D]}\right). \tag{S42}$$

The reaction rate, $r_{2,B\{i\}}$, for $R_{2,B\{i\}}$ is given by,

$$r_{2,B\{i\}} = \nu_D^* \varepsilon_{B\{i\}}^2 \Omega_{2,B\{i\}} [\mathbb{S}_{B\{i\}}] \left(\frac{[\mathrm{C}_D]}{[\mathrm{C}_D] + \kappa^* \varepsilon_{B\{i\}}^4}\right), \text{for } \Delta_r G_{2,B\{i\}} < 0; 0 \text{ otherise.} \tag{S43}$$

Entropy production from reaction $R_{2,B\{i\}}$ and light absorption by $\Omega_{2,B\{i\}}[\mathbb{S}_{B\{i\}}]$ is,

$$\dot{\sigma}_{2,B\{i\}}^R = -\frac{A\zeta_d}{T} r_{2,B\{i\}} \Delta_r G_{2,B\{i\}} \text{ and} \tag{S44}$$

$$\dot{\sigma}_{2,B\{i\}}^P = -\frac{A\zeta_d}{T} \langle I(t) \rangle_d \Delta_r G_\gamma k_p \Omega_{2,B\{i\}} [\mathbb{S}_{B\{i\}}]. \tag{S45}$$

<u>Bacterial decomposition of recalcitrant nitrogen</u>: $R_{3,B\{i\}}$

The reaction rate and entropy production associated with $R_{3,B\{i\}}$ are similar to that for $R_{2,B\{i\}}$, except $R_{3,B\{i\}}$ governs the breakdown of N_D to $\mathrm{NH_3}$ as described by:

$$\mathrm{N}_D \rightarrow \mathrm{NH_3}, \tag{S46}$$

$$\Delta_r G_{3,B\{i\}} = RTln\left(\frac{[\mathrm{NH_3}]}{[\mathrm{N}_D]}\right), \tag{S47}$$

$$r_{3,B\{i\}} = \nu_D^* \varepsilon_{B\{i\}}^2 \Omega_{3,B\{i\}} [\mathbb{S}_{B\{i\}}] \left(\frac{[\mathrm{N}_D]}{[\mathrm{N}_D] + \kappa^* \varepsilon_{Bac}^4}\right), \text{for } \Delta_r G_{3,B\{i\}} < 0; 0 \text{ otherise,} \tag{S48}$$

$$\dot{\sigma}_{3,B\{i\}}^R = -\frac{A\zeta_d}{T} r_{3,B\{i\}} \Delta_r G_{3,B\{i\}}, \tag{S49}$$

$$\dot{\sigma}_{3,B\{i\}}^P = -\frac{A\zeta_d}{T} \langle I(t) \rangle_d \Delta_r G_\gamma k_p \Omega_{3,B\{i\}} [\mathbb{S}_{B\{i\}}]. \tag{S50}$$

S2.2.3 Consumer predation rate, $R_{\chi\{j\},C\{i\}}$

Consumers prey on all function groups including themselves (i.e., cannibalism). We use nearly the same governing equations as before [2], where C in biological structure is converted into biomass, H_2CO_3 and detrital carbon; however, all $C_{P\{i\}}$ storage in phytoplankton is oxidized to H_2CO_3 instead of being excreted as C_L. Excess N and P from consumed biological structure is excreted in both labile and detrital forms as a function of $\varepsilon_{C\{i\}}$. The rational is that when a consumer grows with high thermodynamic efficiency ($\varepsilon_{C\{i\}}$ closer to 1), prey are processed more effectively leading to NH_3 and H_3PO_4 production, while low efficiency growth leads to more detrital products. This version also weights allocation to prey consumption normalized by prey density, as given by $\omega_{\chi\{j\},C\{i\}}$ below; consequently, no constraint is placed on the sum, $\sum_j \Omega_{\chi\{j\},\mathrm{C}\{i\}}$, as it is for phytoplankton and bacteria (in theory, one degree of freedom could be removed though). In the equations below, the subscript $\chi\{j\}$ represented any ecotype, $\{j\}$, of any of the three functional groups, where χ can be P, B or C, and $[\mathrm{C}_{\chi\{j\}}]$ equals 0 when χ is equal to B or C, since those functional groups have no internal carbon storage in this version of the model.

Stoichiometry of reaction $R_{\chi\{j\},C\{i\}}$ and coefficients to balance O and H are given by,

$$\begin{aligned}
&\mathbb{S}_{\chi\{j\}} + \frac{[\mathrm{C}_{\chi\{j\}}]}{[\mathbb{S}_{\chi\{j\}}]}\mathrm{C}_{\chi\{j\}} + \left(a^{C}_{C\{i\}}\left(1-\varepsilon_{C\{i\}}\right) + \frac{[\mathrm{C}_{\chi\{j\}}]}{[\mathbb{S}_{\chi\{j\}}]}\right)\mathrm{O}_2(aq) \\
&\quad \to \varepsilon_{C\{i\}}\mathbb{S}_{C\{i\}} + \left(1-\varepsilon_{C\{i\}}\right)\left(\left(1-\varepsilon_{C\{i\}}\right)\mathrm{H_2CO_3} + \varepsilon_{C\{i\}}\mathrm{C}_D\right) \\
&\quad + \gamma_{\mathbb{S}}\left(1-\varepsilon_{C\{i\}}\right)\left(\left(1-\varepsilon_{C\{i\}}\right)\mathrm{NH_3} + \varepsilon_{C\{i\}}\mathrm{N}_D\right) \\
&\quad + \delta_{\mathbb{S}}\left(1-\varepsilon_{C\{i\}}\right)\left(\left(1-\varepsilon_{C\{i\}}\right)\mathrm{H_3PO_4} + \varepsilon_{C\{i\}}\mathrm{P}_D\right) + b^{C}_{C}\left(1-\varepsilon_{C\{i\}}\right)\mathrm{H_2O} \\
&\quad + \frac{[\mathrm{C}_{\chi\{j\}}]}{[\mathbb{S}_{\chi\{j\}}]}\mathrm{H_2CO_3},
\end{aligned} \tag{S51}$$

$$a^{C}_{C\{i\}} = \tfrac{1}{4}\left(4 + \alpha_{\mathbb{S}} - 2\beta_{\mathbb{S}} - 3\gamma_{\mathbb{S}} + 5\delta_{\mathbb{S}} - 4\varepsilon_{C\{i\}}\right) \text{ and} \tag{S52}$$

$$b^{C}_{C} = \tfrac{1}{2}(-2 + \alpha_{\mathbb{S}} - 3\gamma_{\mathbb{S}} - 3\delta_{\mathbb{S}}). \tag{S53}$$

Standard Gibbs free energy of reaction for $R_{\chi\{j\},C\{i\}}$ is given by,

$$\begin{aligned}
&\Delta_r G^{o}_{\chi\{j\},C\{i\}} \\
&\quad = \Bigg(\varepsilon_{C\{i\}}\Delta_f G^{o}_{\mathbb{S}} + \left(\left(1-\varepsilon_{C\{i\}}\right)^2 + \frac{[\mathrm{C}_{\chi\{j\}}]}{[\mathbb{S}_{\chi\{j\}}]}\right)\Delta_f G^{o}_{H_2CO_3} + \varepsilon_{C\{i\}}\left(1-\varepsilon_{C\{i\}}\right)\Delta_f G^{o}_{C_D} \\
&\quad + \delta_{\mathbb{S}}\left(1-\varepsilon_{C\{i\}}\right)\Delta_f G^{o}_{H_3PO_4} + \gamma_{\mathbb{S}}\left(1-\varepsilon_{C\{i\}}\right)\Delta_f G^{o}_{NH_3} + b^{C}_{C}\left(1-\varepsilon_{C\{i\}}\right)\Delta_f G^{o}_{H_2O}\Bigg) \\
&\quad - \left(\Delta_f G^{o}_{\mathbb{S}} + \left(a^{C}_{C\{i\}}\left(1-\varepsilon_{C\{i\}}\right) + \frac{[\mathrm{C}_{\chi\{j\}}]}{[\mathbb{S}_{\chi\{j\}}]}\right)\Delta_f G^{o}_{O_2(aq)} + \frac{[\mathrm{C}_{\chi\{j\}}]}{[\mathbb{S}_{\chi\{j\}}]}\Delta_f G^{o}_{CH_2O}\right),
\end{aligned} \tag{S54}$$

and the Gibbs free energy of reaction for $R_{\chi\{j\},C\{i\}}$ accounting for reaction quotient is,

$$\begin{aligned}
&\Delta_r G_{\chi\{j\},C\{i\}} \\
&\quad = \Delta_r G^{o}_{\chi\{j\},C\{i\}} + RTln\left(\left[\mathbb{S}_{C\{i\}}\right]^{\varepsilon_{C\{i\}}}[\mathrm{H_2CO_3}]^{\left(1-\varepsilon_{C\{i\}}\right)^2}[\mathrm{C}_D]^{\varepsilon_{C\{i\}}\left(1-\varepsilon_{C\{i\}}\right)}\right) \\
&\quad + RTln\left([\mathrm{NH_3}]^{\gamma_{\mathbb{S}}\left(1-\varepsilon_{C\{i\}}\right)^2}[\mathrm{N}_D]^{\gamma_{\mathbb{S}}\varepsilon_{C\{i\}}\left(1-\varepsilon_{C\{i\}}\right)}\right) \\
&\quad + RTln\left([\mathrm{H_3PO_4}]^{\delta_{\mathbb{S}}\left(1-\varepsilon_{C\{i\}}\right)^2}[\mathrm{P}_D]^{\delta_{\mathbb{S}}\varepsilon_{C\{i\}}\left(1-\varepsilon_{C\{i\}}\right)}\right) \\
&\quad - RTln\left(\left[\mathbb{S}_{\chi\{j\}}\right]\left[\mathrm{C}_{\chi\{j\}}\right]^{\left[C_{\chi\{j\}}\right]/[\chi\{j\}]}[\mathrm{O}_2(aq)]^{a^{C}_{C\{i\}}\left(1-\varepsilon_{C\{i\}}\right)}\right).
\end{aligned} \tag{S55}$$

The electrons transferred in catabolic sub-reaction of $R_{\chi\{j\},C\{i\}}$ is,

$$n^{e}_{C\{i\}} = 4. \tag{S56}$$

Consumer preference for prey $\mathbb{S}_{\chi\{j\}}$, given by $\Omega_{\chi\{j\},C\{i\}}$, is weighted by all prey concentrations that consumer $\mathbb{S}_{C\{j\}}$ is allowed to eat is given by,

$$\omega_{\chi\{j\},C\{i\}} = \frac{\Omega_{\chi\{j\},C\{i\}}\left[\mathbb{S}_{\chi\{j\}}\right]}{\sum_j \Omega_{P\{j\},C\{i\}}\left[\mathbb{S}_{P\{j\}}\right] + \sum_j \Omega_{B\{j\},C\{i\}}\left[\mathbb{S}_{B\{j\}}\right] + \sum_j \Omega_{C\{j\},C\{i\}}\left[\mathbb{S}_{C\{j\}}\right]}. \tag{S57}$$

The reaction rate, $r_{\chi\{j\},C\{i\}}$, of reaction $R_{\chi\{j\},C\{i\}}$ is,

$$\begin{aligned}
&r_{\chi\{j\},C\{i\}} \\
&= \nu^{*}\varepsilon^{2}_{C\{i\}}\omega_{\chi\{j\},C\{i\}}\left[\mathbb{S}_{C\{i\}}\right]\left(\frac{\left[\mathbb{S}_{\chi\{j\}}\right]}{\left[\mathbb{S}_{\chi\{j\}}\right] + \kappa^{*}\varepsilon^{4}_{C\{i\}}}\right)\left(\frac{[\mathrm{O}_2(aq)]}{[\mathrm{O}_2(aq)] + \kappa^{*}\varepsilon^{4}_{C\{i\}}}\right)F_T\left(\Delta_r G_{\chi\{j\},C\{i\}}, n^{e}_{C\{i\}}\right).
\end{aligned} \tag{S58}$$

Entropy production from reaction $R_{\chi\{j\},C\{i\}}$ and light absorption by $\mathbb{S}_{C\{i\}}$ allocated to prey $\chi\{j\}$ is given by,

$$\dot{\sigma}^{R}_{\chi\{j\},C\{i\}} = -\frac{A\zeta_d}{T}r_{\chi\{j\},C\{i\}}\Delta_r G_{\chi\{j\},C\{i\}} \text{ and} \tag{S59}$$

$$\dot{\sigma}^{P}_{\chi\{j\},C\{i\}} = -\frac{A\zeta_d}{T}\langle I(t)\rangle_d \Delta_r G_{\gamma} k_p \Omega_{\chi\{j\},C\{i\}}\left[\mathbb{S}_{C\{i\}}\right]. \tag{S60}$$

S2.3 Reaction Network, $\boldsymbol{S}(\boldsymbol{u})\boldsymbol{r}(t;\boldsymbol{u})$

The reaction network is defined by the stoichiometries of the reactions listed above. Instead of listing all the elements of the stoichiometric matrix, $\mathbf{S}(\mathbf{u})$, which is sparse, we list the n_S rows of the vector that results from the matrix vector product of $\mathbf{S}(\mathbf{u})\mathbf{r}(t;\mathbf{u})$, which is a mass balance around each state variable. For instance, $\mathbf{S}_{\mathbb{S}_{P\{i\}}}^T\mathbf{r}$ is the net production rate of $\mathbb{S}_{P\{j\}}$ resulting from the growth and predation. The rows of $\mathbf{S}(\mathbf{u})\mathbf{r}(t;\mathbf{u})$ are as follows:

$$\begin{aligned}\mathbf{S}_{DIC}^T\mathbf{r} = &\sum_{i=1}^{n_P}\left(-\varepsilon_{P\{i\}}r_{1,P\{i\}} + \left(1+\varepsilon_{P\{i\}}\left(n_{2,P\{i\}}-1\right)\right)r_{2,P\{i\}}\right)\\ &+\sum_{i=1}^{n_B}\left(2-\varepsilon_{B\{i\}}\left(a_{1,B}^A+1\right)\right)r_{1,B\{i\}} + \sum_{i=1}^{n_C}\sum_{j=1}^{n_P}\left(\left(1-\varepsilon_{C\{i\}}\right)^2+\frac{[\mathrm{C}_{P\{j\}}]}{[\mathbb{S}_{P\{j\}}]}\right)r_{P\{j\},C\{i\}}\\ &+\sum_{i=1}^{n_C}\sum_{j=1}^{n_B}\left(1-\varepsilon_{C\{i\}}\right)^2 r_{B\{j\},C\{i\}} + \sum_{i=1}^{n_C}\sum_{j=1}^{n_C}\left(1-\varepsilon_{C\{i\}}\right)^2 r_{C\{j\},C\{i\}},\end{aligned} \tag{S61}$$

$$\begin{aligned}\mathbf{S}_{O_2}^T\mathbf{r} = &\sum_{i=1}^{n_P}\left(\varepsilon_{P\{i\}}r_{1,P\{i\}} - \left(1+\varepsilon_{P\{i\}}\left(a_{2,P}^A+n_{2,P\{i\}}-1\right)\right)r_{2,P\{i\}}\right) - \sum_{i=1}^{n_B}\left(1-\varepsilon_{B\{i\}}\right)r_{1,B\{i\}}\\ &-\sum_{i=1}^{n_C}\sum_{j=1}^{n_P}\left(a_{C\{i\}}^C\left(1-\varepsilon_{C\{i\}}\right)+\frac{[\mathrm{C}_{P\{j\}}]}{[\mathbb{S}_{P\{j\}}]}\right)r_{P\{j\},C\{i\}} - \sum_{i=1}^{n_C}\sum_{j=1}^{n_B}a_{C\{i\}}^C\left(1-\varepsilon_{C\{i\}}\right)r_{B\{j\},C\{i\}}\\ &-\sum_{i=1}^{n_C}\sum_{j=1}^{n_C}a_{C\{i\}}^C\left(1-\varepsilon_{C\{i\}}\right)r_{C\{j\},C\{i\}},\end{aligned} \tag{S62}$$

$$\mathbf{S}_{C_L}^T\mathbf{r} = \sum_{i=1}^{n_B}\left(-r_{1,B\{i\}}+r_{2,B\{i\}}\right), \tag{S63}$$

$$\begin{aligned}\mathbf{S}_{C_D}^T\mathbf{r} = &-\sum_{i=1}^{n_B}r_{2,B\{i\}} + \sum_{i=1}^{n_C}\sum_{j=1}^{n_P}\varepsilon_{C\{i\}}\left(1-\varepsilon_{C\{i\}}\right)r_{P\{j\},C\{i\}} + \sum_{i=1}^{n_C}\sum_{j=1}^{n_B}\varepsilon_{C\{i\}}\left(1-\varepsilon_{C\{i\}}\right)r_{B\{j\},C\{i\}}\\ &+\sum_{i=1}^{n_C}\sum_{j=1}^{n_C}\varepsilon_{C\{i\}}\left(1-\varepsilon_{C\{i\}}\right)r_{C\{j\},C\{i\}},\end{aligned} \tag{S64}$$

$$\begin{aligned}\mathbf{S}_{N_D}^T\mathbf{r} = &-\sum_{i=1}^{n_B}r_{3,B\{i\}} + \sum_{i=1}^{n_C}\sum_{j=1}^{n_P}\gamma_{\mathbb{S}}\varepsilon_{C\{i\}}\left(1-\varepsilon_{C\{i\}}\right)r_{P\{j\},C\{i\}} + \sum_{i=1}^{n_C}\sum_{j=1}^{n_B}\gamma_{\mathbb{S}}\varepsilon_{C\{i\}}\left(1-\varepsilon_{C\{i\}}\right)r_{B\{j\},C\{i\}}\\ &+\sum_{i=1}^{n_C}\sum_{j=1}^{n_C}\gamma_{\mathbb{S}}\varepsilon_{C\{i\}}\left(1-\varepsilon_{C\{i\}}\right)r_{C\{j\},C\{i\}},\end{aligned} \tag{S65}$$

$$\begin{aligned}\mathbf{S}_{NH_3}^T\mathbf{r} = &-\sum_{i=1}^{n_P}\varepsilon_{P\{i\}}\gamma_{\mathbb{S}}r_{2,P\{i\}} + \sum_{i=1}^{n_B}\left(r_{3,B\{i\}}-\varepsilon_{B\{i\}}\gamma_{\mathbb{S}}r_{1,B\{i\}}\right) + \sum_{i=1}^{n_C}\sum_{j=1}^{n_P}\gamma_{\mathbb{S}}\left(1-\varepsilon_{C\{i\}}\right)^2 r_{P\{j\},C\{i\}}\\ &+\sum_{i=1}^{n_C}\sum_{j=1}^{n_B}\gamma_{\mathbb{S}}\left(1-\varepsilon_{C\{i\}}\right)^2 r_{B\{j\},C\{i\}} + \sum_{i=1}^{n_C}\sum_{j=1}^{n_C}\gamma_{\mathbb{S}}\left(1-\varepsilon_{C\{i\}}\right)^2 r_{C\{j\},C\{i\}},\end{aligned} \tag{S66}$$

$$\mathbf{S}_{\mathbb{S}_{P\{i\}}}^T\mathbf{r} = \varepsilon_{P\{i\}}r_{2,P\{i\}} - \sum_{j=1}^{n_C}r_{P\{i\},C\{j\}}, \tag{S67}$$

$$\mathbf{S}^{T}_{C\,P\{i\}}\mathbf{r} = \varepsilon_{P\{i\}} r_{1,P\{i\}} - \left(1 + \varepsilon_{P\{i\}} n_{2,P\{i\}}\right) r_{2,P\{i\}} - \sum_{j=1}^{n_C} \frac{[C_{P\{i\}}]}{[\mathbb{S}_{P\{i\}}]} r_{P\{i\},C\{j\}}, \tag{S68}$$

$$\mathbf{S}^{T}_{\mathbb{S}\,B\{i\}}\mathbf{r} = \varepsilon_{B\{i\}} a^{A}_{1,B} r_{1,B\{i\}} - \sum_{j=1}^{n_C} r_{B\{i\},C\{j\}}, \tag{S69}$$

$$\mathbf{S}^{T}_{\mathbb{S}\,C\{i\}}\mathbf{r} = \sum_{j=1}^{n_P} \varepsilon_{C\{i\}} r_{P\{j\},C\{i\}} + \sum_{j=1}^{n_B} \varepsilon_{C\{i\}} r_{B\{j\},C\{i\}} + \sum_{j=1}^{n_C} \varepsilon_{C\{i\}} r_{C\{j\},C\{i\}} - \sum_{j=1}^{n_C} r_{C\{i\},C\{j\}}. \tag{S70}$$

S2.4 Integrated Entropy Production

Cumulative entropy production over the simulation (or optimization) interval is determined by summing then integrating the contributions of reactions, particle absorptions and water, as given by,

$$\begin{aligned}\sigma^{R} = &\int_{t_0}^{t_f} \sum_{i=1}^{n_P} \left(\dot{\sigma}^{R}_{1,P\{i\}}(\tau) + \dot{\sigma}^{R}_{2,P\{i\}}(\tau)\right) d\tau + \int_{t_0}^{t_f} \sum_{i=1}^{n_B} \left(\dot{\sigma}^{R}_{1,B\{i\}}(\tau) + \dot{\sigma}^{R}_{2,B\{i\}}(\tau) + \dot{\sigma}^{R}_{3,B\{i\}}(\tau)\right) d\tau \\ &+ \int_{t_0}^{t_f} \sum_{j=1}^{n_\chi} \sum_{i=1}^{n_C} \dot{\sigma}^{R}_{\chi\{j\},C\{i\}}(\tau)\, d\tau,\end{aligned} \tag{S71}$$

$$\begin{aligned}\sigma^{P} = &\int_{t_0}^{t_f} \sum_{i=1}^{n_P} \left(\dot{\sigma}^{P}_{1,P\{i\}}(\tau) + \dot{\sigma}^{P}_{2,P\{i\}}(\tau)\right) d\tau + \int_{t_0}^{t_f} \sum_{i=1}^{n_B} \left(\dot{\sigma}^{P}_{1,B\{i\}}(\tau) + \dot{\sigma}^{P}_{2,B\{i\}}(\tau) + \dot{\sigma}^{P}_{3,B\{i\}}(\tau)\right) d\tau \\ &+ \int_{t_0}^{t_f} \sum_{j=1}^{n_\chi} \sum_{i=1}^{n_C} \dot{\sigma}^{P}_{\chi\{j\},C\{i\}}(\tau) d\tau \text{ and}\end{aligned} \tag{S72}$$

$$\sigma^{W} = \int_{t_0}^{t_f} \dot{\sigma}^{W}(\tau) d\tau. \tag{S73}$$

The total entropy production used in the optimization described below is simply the sum,

$$\sigma^{T} = \sigma^{R} + \sigma^{W} + \sigma^{P}. \tag{S74}$$

S2.5 Initial Value Problem Integration

The numerical package BiM [8], which uses blended implicit methods to integrate stiff ordinary differential equations (ODEs), was used to solve the initial value problem (Eqn. S1) and to determine cumulative entropy production, Eqn. (S71-S74). All simulations were run for two years, 10^{-6} was used for both absolute and relative tolerances, a maximum step size (*hmax*) of 0.05 was implemented to insure diel light cycles were not stepped over, *maxstep* was increased to 10000000, and finite differences were used to calculate the Jacobian matrix. Default values were used for all other BiM options.

S3 Optimization of Trait-Based Model

Instead of employing optimal control to determine how $\varepsilon_{\chi\{i\}}$ and $\Omega_{j,\chi\{i\}}$ vary over time as has been previously used [2,9], in this manuscript we investigated a hybrid between MEP optimization and trait-based modeling. In typical trait-based models [1], a large number of each functional group are included in the model, and the traits, (i.e., $\varepsilon_{\chi\{i\}}$ and $\Omega_{j,\chi\{i\}}$) are randomly assigned values. When a simulation is run, organisms that grow fastest under the prevailing simulated environment dominate, while others are effectively culled from the population in a manner analogous to natural selection, but *in silico*. In order to explore the trait space, a large population of each functional group is needed; however, this presents a problem in our current model formulation. As the population size of $\mathbb{S}_P$ and $\mathbb{S}_B$ are increased, the column dimension of the predation matrix, $\Omega_{\chi\{j\},C\{i\}}$, increases, so that the total number of traits in the model, given by $n_T = 2n_P + 3n_B + (1 + n_P + n_B + n_C)n_C$, increases rapidly with population size. For instance, a model with just 10 ecotypes in each functional group has 360 trait values in total, and one with 100 ecotypes each has 30,600 trait values in total; the size of the trait space scales with $O(n^2)$. One way to circumvent the scaling problem is to limit the number of prey each consumer can target, but this places more constraints on the structure of the food web than we desired. While we investigated the standard trait-based approach, we found the $O(n^2)$ scaling on trait space made the approach untenable for our objectives; consequently, we developed a new, hybrid approach.

Instead of randomly assigning trait values, the hybrid approach numerically searches for trait values that maximize the objective function, in this case total entropy production. This approach does not require a large number of ecotypes of each functional group, because the trait space is not being explored randomly, but systematically using optimization. Even simulations with just one instance of each functional group $(n_T = 9)$ generated reasonable solutions. In fact, as discussed in the main text, adding food web complexity in the form of more ecotypes did significantly increase EP in many of our 0D simulations. The hybrid approach differs from the optimal control approach in that neither $\varepsilon_{\chi\{i\}}$ nor $\Omega_{j,\chi\{i\}}$ vary during a simulation. Consequently, the size/complexity of the food web likely needs to be larger for temporally and spatially varying environments in order to cover all niches, but time varying environments, other than light intensity, were not invested in this study. Once optimal parameter values are determined for a given set of environmental conditions, the optimization component does not need to be rerun.

S3.1 hyperBOB

For the optimization, we used the derivative-free, box-constrained, local optimizer BOBYQA[10] to search the n_T dimensional trait space by maximizing σ^T defined by Eqns. (S71-S74). To search for a global optimum on a computer cluster with N_{CPU} CPUs, BOBYQA was started with N_{CPU} different initial conditions that were selected by sampling from a Latin unit hypercube [11], which we implemented in the routine hyperBOB (DOI: 10.5281/zenodo.3978689). Parameters used in BOBYQA/hyperBOB were: *rhobeg*, 0.49; *rhoend*, 0.0001; *maxfun*, 10,000. Except on rare occasions, solutions were found before the maximum number of function calls (*maxfun*) occurred. A time constraint was also placed on the solution, but it also seldom was invoked. All simulations were run on a 5-node computer cluster with a total of 90 CPU cores.

S3.2 Temporal Strategies for Phytoplankton

To investigate how temporal strategies, in particular circadian rhythms, increase entropy production, we used two different approaches. In the first approach that was later retired, the constant trait value assigned to $\Omega_{1,P\{i\}}$ was replaced by a time varying function that depends to two new trait variables, $f_{P\{i\}}$ and $\varphi_{P\{i\}}$, as given by,

$$\Omega_{1,P\{i\}}(t) = \frac{1}{2}\left(\sin\left(2\pi f_{P\{i\}}t + \varphi_{P\{i\}}\right) + 1\right), \tag{S75}$$

where $f_{P\{i\}}$ is the frequency $[\![d^{-1}]\!]$ and $\varphi_{P\{i\}}$ the phase $[\![rad]\!]$ of $\Omega_{1,P\{i\}}(t)$ that controls allocation of phytoplankton protein to CO_2 fixation given by reaction $R_{1,P\{i\}}$. When $f_{P\{i\}} = 0$, $\varphi_{P\{i\}}$ modifies the amplitude of $\Omega_{1,P\{i\}}$, but does not change over time. These two traits had the following bounds,

$$0 \le f_{P\{i\}} \le 2 \text{ and } 0 \le \varphi_{P\{i\}} \le 2\pi. \tag{S76}$$

Several simulation studies were conducted with the above temporal modification of $\Omega_{1,P\{i\}}(t)$; however, by allowing the frequency parameter to be a trait variable, we found that locating the global optimum proved challenging as evident in Figure S1, in which all traits where held constant for a $1P1B1C$ simulation and σ^R was calculated for different values of $f_{P\{1\}}$ and $\varphi_{P\{1\}}$ at high resolution (1051 uniform samples in each dimension). Even though local optima occur for other frequencies, all simulations investigated showed the global optimum only occurred for $f_{P\{1\}} = 1\ d^{-1}$; consequently, we used a different function for $\Omega_{1,P\{i\}}(t)$ in which frequency was fixed to the diel cycle of 1 per day to improve computational speed.

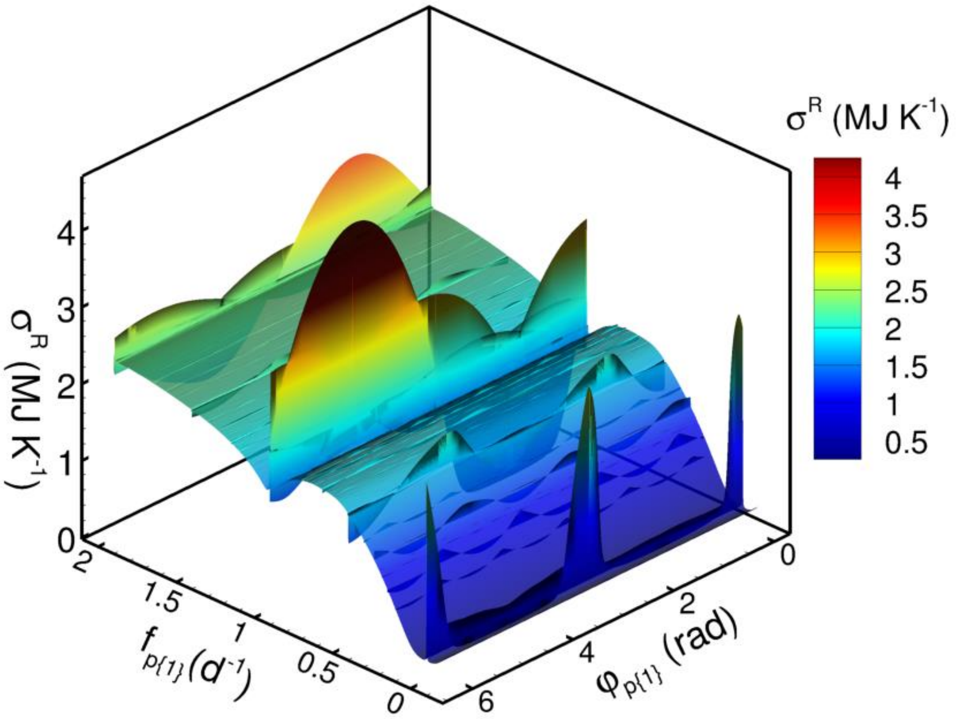

Figure S1. Reaction entropy production, σ^R, for different values of $f_{P\{1\}}$ and $\varphi_{P\{1\}}$ for Eqn. (S75) while all other traits held constant for a $1P1B1C$ food web model. Note, the global optimum is located at a frequency of 1 d^{-1}; however, the peak is very narrow and was often missed by the hyperBOB search algorithm unless constraints on $f_{P\{1\}}$ were centered near 1.

For all simulations described in the main text, the following time varying square wave function for $\Omega_{1,P\{i\}}(t)$ was used instead of Eqn. (S75),

$$\Omega_{1,P\{i\}}(t) = \Omega_{amp\{i\}} \max\left(\delta_S\left(\text{mod}(t,1), t_{On\{i\}}, \lambda_s\right) + \delta_S\left(\text{mod}(t,1), t_{Off\{i\}}, -\lambda_s\right) - 1, 0\right), \tag{S77}$$

where $\delta_S(t, t_s, \lambda_s)$ is a smooth unit step function around t_s given by,

$$\delta_S(t, t_s, \lambda_s) = \frac{1}{e^{-\lambda_s(t-t_s)} + 1}. \tag{S78}$$

In Eqn. (S77), three parameters, all bounded between 0 and 1, govern the characteristics of the $\Omega_{1,P\{i\}}(t)$ square-wave step function that occurs each day: $t_{On\{i\}}$ specifies time of day when the step-up occurs; $t_{Off\{i\}}$ specifies when the step-down occurs; $\Omega_{amp\{i\}}$ specifies the amplitude of the step. The parameter λ_s was not considered a trait, but rather is used to control numerical smoothness of the step and was set to a value of 200 d^{-1} for all simulations. For either Eqn. (S75) or (S77), since biomass allocation must be conserved, $\Omega_{2,P\{i\}}(t)$ is obtained from the difference given by $1 - \Omega_{1,P\{i\}}(t)$.

One of the three types of temporal strategies discussed in the main text examines the impact of strict balanced growth for phytoplankton, so that the C:N ratio of phytoplankton remains constant (technically, this is a no-temporal-strategy strategy). To achieve balanced growth, $r_{1,P\{i\}}$ must be coupled to $r_{2,P\{i\}}$ so that the ratio of $\mathbb{S}_{P\{i\}}$ concentration to $C_{P\{i\}}$ concentration remains constant, or that,

$$\frac{d\,{}^{[C_{P\{i\}}]}/_{[\mathbb{S}_{P\{i\}}]}}{dt} = 0, \tag{S79}$$

which occurs when,

$$\frac{r_{1,P\{i\}}(t)}{r_{2,P\{i\}}(t)} = \beta_{P\{i\}} \stackrel{\text{def}}{=} \left(\frac{1}{\varepsilon_{P\{i\}}} - n_{2,P\{i\}} + k_{PP\{i\}}\right), \tag{S80}$$

where $k_{PP\{i\}}$ is a specified constant that sets the ratio's $[C_{P\{i\}}]/[\mathbb{S}_{P\{i\}}]$ value, which was set to 1 for all balance growth simulations. In simulations with balanced growth (i.e., no temporal strategy), $r_{1,P\{i\}}(t)$ and $r_{2,P\{i\}}(t)$ are calculated based on Eqns. (S12) and (S26), respectively, then adjusted as follows,

$$r_{1,P\{i\}}(t) = \min\left(r_{1,P\{i\}}(t), \beta_{P\{i\}} r_{2,P\{i\}}(t)\right) \text{ and} \tag{S81}$$

$$r_{2,P\{i\}}(t) = \min\left(r_{2,P\{i\}}(t), \frac{1}{\beta_{P\{i\}}} r_{1,P\{i\}}(t)\right). \tag{S82}$$

For example, at night, $r_{1,P\{i\}}(t)$ is zero and Eqn. (S82) forces $r_{2,P\{i\}}(t)$ to zero. Similarly, if no NH_3 is present, so that $r_{2,P\{i\}}(t)$ equals zero, then $r_{1,P\{i\}}(t)$ is set to zero based on Eqn. (S81) and phytoplankton dissipate solar radiation as particles, as described in Section S2.2.1 above.

Simulations in the main text were conducted with version 4.7 of the model, which can be obtained from GitHub (DOI: 10.5281/zenodo.3979922).

S4 Example parameter input file

Below is the parameter file used for the circadian clock strategy at a dilution rate of 0.2 d^{-1} using the nominal input concentrations given in Table 2 of the main text.

```
! Run149_opt4.5_1p1b1c
! Using AutoHetDet_Opt_V4.5
! 16-Jun-2020 on MEP
```

```
! This is Run149, but using V4.5.
! three parameters to specify a square wave function for omg_pp

&params
! Input parameters
npp   = 1 ! number of S1 primary producers
nbac  = 1 ! number of bacteria
ncc   = 1 ! number of S2 consumers

! sumSigWeights determines which EP terms to use for optimization.
! Weights on EP where vector is: [Rxns, H20, particles]
! Total EP production use 1., 1., 1., for just rxn, use 1., 0., 0., etc.
sumSigWeights = 1., 1., 1.

! These parameters are used for EP surface generation only.
genSurf = .false. ! Should an EP surface be generated instead of optimization
readeps = .false. ! Read in the trait values from file.
whichPP = 1 ! which of the possible pp's to run f_pp and phi_pp over
nSurfPts = 1051  ! Number of points in the x and y dimension of the 2D surface to produce
reportTime = 10. ! How often to update screen during problem (min).

iseed = 10 ! changing this value to produce a different set of random values.
nuStar = 350.  ! Used in adaptive Monod equation 1/d
nuDet  = 175. ! For detritus decomp (1/d)
kappa = 5000. ! Adaptive Monod equation universal parameter (uM)
surA = 1.0 ! surface area of pond (m^2).
T_K = 293. ! get temperature (K)
pH  = 8.1  ! pH
depth = 1.0 ! Pond depth (m)
is = 0.72  ! Ionic strength (M) = 0.72*sal/35.0 (sal is salinity (PSU))
dil_t0 = 0.2 ! dilution rate at t0 (1/d)
dil_tf = 0.2 ! dilution rate at tf (1/d)
dil_n = 0    ! number of steps in dilution rate between t0 and tf

! Parameters associated with in-silico selection of traits
minCompFac = 500000.0 ! If process takes longer than (tf-t0)/minCompFac, then terminate
epp_min = 0.00001 ! min and max values for epp
epp_max = 1.0
ecc_min = 0.00001 ! min and max values for ecc
ecc_max = 1.0
ebac_min = 0.0001 ! min and max values for ebac
ebac_max = 1.0

! limits and parameters
! The square wave is limited to occur every day, so frequency is fixed in V4.0 and later
! tOn_pp is when the step up occurs, and tOff_pp when steps down occurs. These are in days.
! Note, the overhangs (< 0 on tOn and >1 on tOff) insures omg_pp can be fully on all day
! because of the nature of the exp step function and value of sigOmg_pp.
! These are used for circadian strategy
sigOmg_pp = 200. ! this is used in the exp setup function to make a "smooth" square wave
tOn_pp_min  = -0.05 ! lower limit on on time (d)
tOn_pp_max  = 1.0   ! upper limit when step up can occur (d)
tOff_pp_min = 0.0   ! lower limit when a step down can occur (d)
tOff_pp_max = 1.05  ! upper limit when step down occurs (d)
```

```
! Use these for no circadian rhythm (i.e., passive storage).
! tOn_pp_min  = -0.05 ! lower limit on on time (d)
! tOn_pp_max  = -0.049   ! upper limit when step up can occur (d)
! tOff_pp_min = 1.04   ! lower limit when a step down can occur (d)
! tOff_pp_max = 1.05  ! upper limit when step down occurs (d)

! V4.7 Add binaryOMG to set omg_cc to binary matrix (only 0's or 1's)
binaryOMG = .false. ! default is .false.

! Coupling between r_1,p and r_2,p.  If k_pp below is set to zero (default) then
! reactions are not coupled, but if k_pp > 0, then it sets the ratio of
! C_p to p (i.e., C_p/p = k_pp). Note, there is also the variable k_p for light
! attenuation that is different. When k_pp /= 0, tOn_pp and tOff_pp should be set to
! the passive storage scenario.
k_pp = 0.0

! Initial and feed concentrations in input feed. All concentrations in uM
dicI = 2000. ! (uM)
o2I = 225. ! (uM)
nh3I_t0 = 5. ! nh3I at t0 (uM)
nh3I_tf = 5. ! nh3I at tf (uM)
nh3I_n  = 0   ! Number of steps in nh3I between t0 and tf
c_LI = 10. ! labile carbon (uM)
c_dI = 100.0 ! detrital carbon (uM)
n_dI = 7. ! detrital nitrogen (uM)
ppI   = 0.1 ! initialize all phytoplankton to this value (uM)
c_ppI = 0.1 ! all phytoplankton carbon stores (uM)
ccI   = 0.1 ! initialize all consumers to this value (uM)
bacI  = 0.1 ! all bacteria (uM)

! Phosphate concentrations. Held fixed, but used for thermodynamic calculations
h3po4 = 1. ! uM
P_d   = 5. ! detrital P (uM)

! Biomass elemental composition. From Battley1998 for yeast. Unit carbon
alf = 1.613 ! H
bet = 0.557 ! O
gam = 0.158 ! N
del = 0.012 ! P
cell_F = 1000.0 ! concentration factor for C_P. Intracellular versus extracellular volume.
delPsi = 0.1 ! LaRowe's thermo driver.  Cell membrane potential (V)

! Gas Exchange, temp and pH
pV_o2  = 3.0 ! piston velocity for O2 (m/d)
pV_co2 = 2.6 ! piston velocity for CO2 (m/d)
pO2    = 0.21    ! partial pressure of O2 in atmosphere (atm)
pCO2   = 400.d-6 ! partial pressures CO2 in the atmosphere (atm)

! Solar parameters
I0max = 406000. ! Solar constant in PAR (mmol photons /m^2 /d)
dLat = 42.0 ! Latitude for calculating solar radiation
dGr_Ggamma = -253. ! Gibbs free energy of photons (J/mmol photon of blue light, 440 nm)
k_w = 0.011  ! water attenuation coef (1/m)  (see Table 2.11 of Wozniak2007 for 430 nm light)
k_p = 0.000625 ! attenuation coef by non-algal parties  (m^2/mmol-S)
```

```
k_chla = 0.0025 ! attenuation coef by algal pigments. (Wozniak2007, adjusted to (m^2/mmol-S))

! BiM ODE solution parameters
ompThreads = 1 ! Specify how many threads to use (no currently used, set to 1)
t0 = 0. ! Start time for ODE integration (d)
tDays = 730. ! number of days to run simulation (d)
t0_ep = 0.   ! For optimization, interval over which EP production is maximized.
tf_ep = 730.  ! end of EP interval.
maxstep_BiM = 10000000 ! maximum number of BiM iterations (set to 0 to use default of 100000)
useOmpJac = 0 ! set to 0 to have BiM calculate numerical gradient
maxattempts = 1 ! number of attempts to solve ODEs before declaring failure
absZero = 1.e-8 ! Numbers less than this are set to this value smoothly. (div by 0 prevention)
atol1 = 1.0e-6  ! absolute tolerance for BiM
rtol1 = 1.0e-6  ! relative tolerance for BiM
hmax_BiM = 0.05 ! largest step size (d). default = (TEND-T0)/8

! parameters used by hyperBOB
rhobeg = 0.49 ! initial and final values of a trust region radius
rhoend = 0.0001 ! When trust region is less than rhoend, stop.
iprint = 0 ! controls amount of printing (0, 1, 2 or 3)
maxfun = 10000 ! maximum number of calls to CALFUN
optimize = .true. ! If true, MEP optimization occurs, otherwise just solve ODEs
fcnUpdate = 100 ! output current status after every fcnUpdate ODE integrations
/
```

S5 References

1. Follows, M.J.; Dutkiewicz, S.; Grant, S.; Chisholm, S.W. Emergent Biogeography of Microbial Communities in a Model Ocean. *Science* **2007**, *315*, 1843-1846, doi:10.1126/science.1138544.
2. Vallino, J.J.; Huber, J.A. Using Maximum Entropy Production to Describe Microbial Biogeochemistry Over Time and Space in a Meromictic Pond. *Frontiers in Environmental Science* **2018**, *6*, 1-22, doi:10.3389/fenvs.2018.00100.
3. Brock, T.D. Calculating solar radiation for ecological studies. *Ecol.Model.* **1981**, *14*, 1-19.
4. Alberty, R.A. *Thermodynamics of biochemical reactions*; Wiley & Sons: Hoboken, NJ, 2003.
5. LaRowe, D.E.; Dale, A.W.; Amend, J.P.; Van Cappellen, P. Thermodynamic limitations on microbially catalyzed reaction rates. *Geochimica et Cosmochimica Acta* **2012**, *90*, 96-109, doi:10.1016/j.gca.2012.05.011.
6. Wozniak, B.; Dera, J. *Light absorption in sea water*; Springer: 2007; Vol. 33, pp. 463.
7. Candau, Y. On the exergy of radiation. *Solar Energy* **2003**, *75*, 241-247, doi:10.1016/j.solener.2003.07.012.
8. Brugnano, L.; Magherini, C. The BiM code for the numerical solution of ODEs. *Journal of Computational and Applied Mathematics* **2004**, *164-165*, 145-158, doi:10.1016/j.cam.2003.09.004.
9. Vallino, J.J.; Algar, C.K.; González, N.F.; Huber, J.A. Use of Receding Horizon Optimal Control to Solve MaxEP-Based Biogeochemistry Problems. In *Beyond the Second Law - Entropy production and non-equilibrium systems*, Dewar, R.C., Lineweaver, C.H., Niven, R.K., Regenauer-Lieb, K., Eds. Springer Berlin Heidelberg: 2014; 10.1007/978-3-642-40154-1_18pp. 337-359.
10. Powell, M.J. *The BOBYQA algorithm for bound constrained optimization without derivatives*; Cambridge NA Report NA2009/06; University of Cambridge: Cambridge, 2009; pp 26-46.

11. McKay, M.D.; Beckman, R.J.; Conover, W.J. A Comparison of Three Methods for Selecting Values of Input Variables in the Analysis of Output from a Computer Code. *Technometrics* **1979**, *21*, 239-245, doi:10.2307/1268522.

© 2020 by the authors. Submitted for possible open access publication under the terms and conditions of the Creative Commons Attribution (CC BY) license (http://creativecommons.org/licenses/by/4.0/).
